# Therapeutic Monitoring of Orally Administered, Small-Molecule Anticancer Medications with Tumor-Specific Cellular Protein Targets in Peripheral Fluid Spaces—A Review

**DOI:** 10.3390/pharmaceutics15010239

**Published:** 2023-01-10

**Authors:** Zoltán Köllő, Miklós Garami, István Vincze, Barna Vásárhelyi, Gellért Balázs Karvaly

**Affiliations:** 1 Department of Laboratory Medicine, Semmelweis University, 1089 Budapest, Hungary; 2 2nd Department of Pediatrics, Semmelweis University, 1094 Budapest, Hungary

**Keywords:** oral anticancer drugs, oncology, imatinib, precision pharmacotherapy, therapeutic drug monitoring, cerebrospinal fluid

## Abstract

Orally administered, small-molecule anticancer drugs with tumor-specific cellular protein targets (OACD) have revolutionized oncological pharmacotherapy. Nevertheless, the differences in exposure to these drugs in the systemic circulation and extravascular fluid compartments have led to several cases of therapeutic failure, in addition to posing unknown risks of toxicity. The therapeutic drug monitoring (TDM) of OACDs in therapeutically relevant peripheral fluid compartments is therefore essential. In this work, the available knowledge regarding exposure to OACD concentrations in these fluid spaces is summarized. A review of the literature was conducted by searching Embase, PubMed, and Web of Science for clinical research articles and case reports published between 10 May 2001 and 31 August 2022. Results show that, to date, penetration into cerebrospinal fluid has been studied especially intensively, in addition to breast milk, leukocytes, peripheral blood mononuclear cells, peritoneal fluid, pleural fluid, saliva and semen. The typical clinical indications of peripheral fluid TDM of OACDs were (1) primary malignancy, (2) secondary malignancy, (3) mental disorder, and (4) the assessment of toxicity. Liquid chromatography–tandem mass spectrometry was most commonly applied for analysis. The TDM of OACDs in therapeutically relevant peripheral fluid spaces is often indispensable for efficient and safe treatments.

## 1. Introduction

The past two decades have seen the rise of a new era of targeted oncological pharmacotherapy. The novel treatment options have led to a tremendous increase in success rates since the first market approval of the now generic imatinib (Gleevec^®^, 2001), an inhibitor of the BCR-ABL oncogenic tyrosine kinase protein, and the first representative of orally administered, small-molecule anticancer drugs with specific tumor-associated cellular protein targets (OACDs). These synthetic molecules bind to proteins that are expressed excessively or even exclusively in cancer cells, resulting in the inhibition of the functions of cancer cells with a limited impact on non-malignant cells. Most OACDs are found in the subgroup L01E of the Anatomical Therapeutic Chemical (ATC) classification system (level 1: “Antineoplastic and immunomodulating agents”, level 2: “Antineoplastic agents”, level 3: “Protein kinase inhibitors”), and are further classified at level 4 as BCR-ABL tyrosine kinase inhibitors, epidermal growth factor receptor (EGFR) tyrosine kinase inhibitors, B-raf serine-threonine kinase (BRAF) inhibitors, anaplastic lymphoma kinase (ALK) inhibitors, mitogen-activated protein kinase (MEK) inhibitors, cyclin-dependent kinase (CDK) inhibitors, mammalian target of rapamycin (mTOR) kinase inhibitors, human epidermal growth factor receptor 2 (HER2) tyrosine kinase inhibitors, Janus-associated kinase (JAK) inhibitors, vascular endothelial growth factor receptor (VEGFR) tyrosine kinase inhibitors, Bruton’s tyrosine kinase (BTK) inhibitors, phosphatidylinositol-3-kinase (Pi3K) inhibitors, fibroblast growth factor receptor (FGFR) tyrosine kinase inhibitors, and “other” protein kinase inhibitors. Further, four OACDs are listed under level 3 code “Other antineoplastic agents” (ATC code: L01X) including histone deacetylase (HDAC) inhibitors, hedgehog pathway inhibitors, and poly-ADP-ribose polymerase inhibitors.

The changes in the treatment of malignancies brought about by OACDs have been revolutionary, considering their favorable adverse effect profiles and applicability as a regular pill medication. Indeed, targeted therapies with OACDs offer significant benefits to patients, clinicians, and the healthcare system with reduced treatment costs, milder and more tolerable adverse effects, and improved prognoses [1]. The range of malignancies that have been treated successfully keeps increasing, with regulatory agencies having granted approvals to over 80 OACDs for treating various types of cancers including central nervous system (CNS) tumors, hematological malignancies, gastrointestinal tumors, and melanoma, as well as non-small cell lung carcinoma (NSCLC), since 2001 [1,2].

Targeted oral anticancer therapy has also brought along new challenges. OACDs are most often administered in one-size-fits-all doses. Nevertheless, the remarkable inter-individual variability in their pharmacokinetic properties raises the need for the individualization of OACD regimens based on the monitoring of drug exposure [3]. Therapy adherence also influences the outcome of the treatment [1]. An increasing number of publications suggests that therapeutic drug monitoring (TDM), as well as the pharmacokinetic interpretation of TDM results, have key importance in optimizing targeted oncotherapy using OACDs [4,5]. The first consensus guideline regarding the TDM of an OACD was published on imatinib in 2021 [6].

The appearance OACDs in physiological or pathologically formed extravascular fluid compartments and in excreta has been demonstrated to bear fundamental clinical relevance. “Peripheral fluid space” is used in the current work to describe fluid compartments in which the repeated measurement of OACD concentrations bears direct therapeutic or toxicological relevance, either because they represent the availability of the drug at the site of the desired or undesired effect, or because the pathological formation of the fluid compartment as a third space alters the availability of the drug in an unpredictable manner. Two exemptions are made. Urine is a physiological excretion end product from which OACDs are not reabsorbed, presenting a fraction no longer biologically available. The appearance of OACDs in amniotic fluid may be informative, but the monitoring of drug levels is unlikely to be associated with any changes in the mater’s medical care, while amniocentesis cannot be viewed as a potential intervention for optimizing OACD therapy. Therefore, we do not recommend the consideration of urine and amniotic fluid as therapeutically relevant peripheral fluid spaces in this context.

Two clinical situations highlight the need for a paradigm shift in the administration of OACDs involving their monitoring in these fluid spaces. First, reports have consistently shown that the amounts of several OACDs which pass through the blood–brain barrier are extremely low. This often leads to failure in treating CNS malignancies in spite of the attainment of sufficient systemic drug exposure [7]. Second, breastfeeding women diagnosed with malignant disorders have been observed to pass on relatively large amounts of the OACD and its metabolites with their breast milk, resulting in unknown biological effects in the lactated infant [8,9,10]. While the approved full prescribing information documents of several OACDs advise women not to breastfeed their infants while taking the medication, the prescription labels are neither categorical, nor consistent in this respect. Overall, it is rational to assume that, in several clinical cases, TDM- and pharmacokinetics-based therapeutic strategies will have to target exposure to OACDs in these peripheral spaces.

To facilitate further research, the aims of this review are (1) to provide a comprehensive overview of the available knowledge regarding the distribution of OACDs to the peripheral fluid spaces, and (2) to explore the methodological approaches employed for the clinical monitoring of the concentrations of OACDs in peripheral fluid spaces.

## 2. Materials and Methods

Due to the types of publications available, a systematic review could not be conducted; however, adhered to the applicable items of the PRISMA Guidelines [11]. The review was not registered. Only substances with per os formulations authorized for human use, identified specific oncogenic cellular protein targets, and a molecular weight not exceeding 1500 Da were assessed. The range of drugs covered is listed under the level 3 ATC code L01E, and under the level 4 codes L10XH, L10XJ, L10XK, and L10XX (as of 12 December 2022, Table 1).

### 2.1. Database Search

A search of the following databases was performed:

1. The database of the National Library of Medicine, National Center for Biotechnology Information (National Library of Medicine, Bethesda, MD, USA, https://pubmed.ncbi.nlm.nih.gov), keyword combination: (abemaciclib OR acalabrutinib OR afatinib OR alectinib OR alpelisib OR anagrelide OR asciminib OR avapritinib OR axitinib OR belzutifan OR binimetinib OR bosutinib OR brigatinib OR cabozantinib OR capmatinib OR ceritinib OR cobimetinib OR copanlisib OR crizotinib OR dabrafenib OR dacomitinib OR dasatinib OR duvelisib OR encorafenib OR entrectinib OR erdafitinib OR erlotinib OR everolimus OR fedratinib OR gefitinib OR gilteritinib OR glasdegib OR ibrutinib OR icotinib OR idelalisib OR imatinib OR infigratinib OR ivosidenib OR ixazomib OR lapatinib OR larotrectinib OR lenvatinib OR lorlatinib OR midostaurin OR mitotane OR neratinib OR nilotinib OR nintedanib OR niraparib OR olaparib OR osimertinib OR pacritinib OR palbociclib OR panobinostat OR pazopanib OR pemigatinib OR pexidartinib OR ponatinib OR pralsetinib OR regorafenib OR ribociclib OR ripretinib OR rucaparib OR ruxolitinib OR selinexor OR selpercatinib OR selumetinib OR sonidegib OR sorafenib OR sotorasib OR sunitinib OR talazoparib OR tazemetostat OR tepotinib OR tivozanib OR trametinib OR tucatinib OR vandetanib OR vemurafenib OR venetoclax OR vismodegib OR vorinostat OR zanubrutinib OR tyrosine kinase inhibit OR PARP) AND (saliva OR cerebrospinal fluid OR liquor OR pleural effusion OR peritoneal dialysis OR interstitial fluid OR brain microdialysis OR semen OR follicular fluid OR tear OR breast milk OR milk OR mother’s milk) AND (concentration OR chromatography OR mass spectrometry OR therapeutic drug monitoring OR levels).

2. The Web of Science (Clarivate^TM^, Chandler, AZ, USA, https://webofscience.com) keyword combination: (abemaciclib OR acalabrutinib OR afatinib OR alectinib OR alpelisib OR anagrelide OR asciminib OR avapritinib OR axitinib OR belzutifan OR binimetinib OR bosutinib OR brigatinib OR cabozantinib OR capmatinib OR ceritinib OR cobimetinib OR copanlisib OR crizotinib OR dabrafenib OR dacomitinib OR dasatinib OR duvelisib OR encorafenib OR entrectinib OR erdafitinib OR erlotinib OR everolimus OR fedratinib OR gefitinib OR gilteritinib OR glasdegib OR ibrutinib OR icotinib OR idelalisib OR imatinib OR infigratinib OR ivosidenib OR ixazomib OR lapatinib OR larotrectinib OR lenvatinib OR lorlatinib OR midostaurin OR mitotane OR neratinib OR nilotinib OR nintedanib OR niraparib OR olaparib OR osimertinib OR pacritinib OR palbociclib OR panobinostat OR pazopanib OR pemigatinib OR pexidartinib OR ponatinib OR pralsetinib OR regorafenib OR ribociclib OR ripretinib OR rucaparib OR ruxolitinib OR selinexor OR selpercatinib OR selumetinib OR sonidegib OR sorafenib OR sotorasib OR sunitinib OR talazoparib OR tazemetostat OR tepotinib OR tivozanib OR trametinib OR tucatinib OR vandetanib OR vemurafenib OR venetoclax OR vismodegib OR vorinostat OR zanubrutinib OR ’tyrosine kinase inhibit’ OR PARP) AND (saliva OR ’cerebrospinal fluid’ OR liquor OR ’pleural effusion’ OR ’peritoneal dialysis’ OR ’interstitial fluid’ OR ’brain microdialysis’ OR semen OR follicular fluid OR tear OR breast milk) AND (concentration OR chromatography OR ’mass spectrometry’ OR ’therapeutic drug monitoring’ OR levels).

3. The Embase database (Elsevier B.V., Amsterdam, The Netherlands, https://embase.com), keyword combination: (abemaciclib OR acalabrutinib OR afatinib OR alectinib OR alpelisib OR anagrelide OR asciminib OR avapritinib OR axitinib OR belzutifan OR binimetinib OR bosutinib OR brigatinib OR cabozantinib OR capmatinib OR ceritinib OR cobimetinib OR copanlisib OR crizotinib OR dabrafenib OR dacomitinib OR dasatinib OR duvelisib OR encorafenib OR entrectinib OR erdafitinib OR erlotinib OR everolimus OR fedratinib OR gefitinib OR gilteritinib OR glasdegib OR ibrutinib OR icotinib OR idelalisib OR imatinib OR infigratinib OR ivosidenib OR ixazomib OR lapatinib OR larotrectinib OR lenvatinib OR lorlatinib OR midostaurin OR mitotane OR neratinib OR nilotinib OR nintedanib OR niraparib OR olaparib OR osimertinib OR pacritinib OR palbociclib OR panobinostat OR pazopanib OR pemigatinib OR pexidartinib OR ponatinib OR pralsetinib OR regorafenib OR ribociclib OR ripretinib OR rucaparib OR ruxolitinib OR selinexor OR selpercatinib OR selumetinib OR sonidegib OR sorafenib OR sotorasib OR sunitinib OR talazoparib OR tazemetostat OR tepotinib OR tivozanib OR trametinib OR tucatinib OR vandetanib OR vemurafenib OR venetoclax OR vismodegib OR vorinostat OR zanubrutinib OR tyrosine kinase inhibit OR PARP) AND (saliva OR cerebrospinal fluid OR liquor OR pleural effusion OR peritoneal dialysis OR interstitial fluid OR brain microdialysis OR semen OR follicular fluid OR tear OR breast milk OR milk) AND (concentration OR chromatography OR mass spectrometry OR therapeutic drug monitoring OR levels).

Scientific works published between 10 May 2001 and 31 August 2022 were evaluated. Since, to the best of the authors’ knowledge, no reviews have been previously written in the same topic, the searched time range was selected to cover the entire period OACDs have been available on the market. No filtering or limiting settings were applied. In the Embase and Web of Science databases, the search was conducted in the titles and in the abstracts (“Title or Abstract”).

In addition, a manual Google search was conducted using the following query terms: “name of drug” AND “therapeutic drug monitoring”, “name of drug” + “milk”, “name of drug” + “liquor”, “name of drug” + “cerebrospinal fluid”, “name of drug” + “semen”, and “name of drug” + “liquid chromatography mass spectrometry”. 

Each database record was evaluated by two reviewers (Z.K. and G.B.K.) who also conducted the manual research. Duplicate publications were removed by Z.K. before screening. No automation tools were employed for evaluating the eligibility of the records.

### 2.2. Screening Eligible Database Records

**Figure 1:**
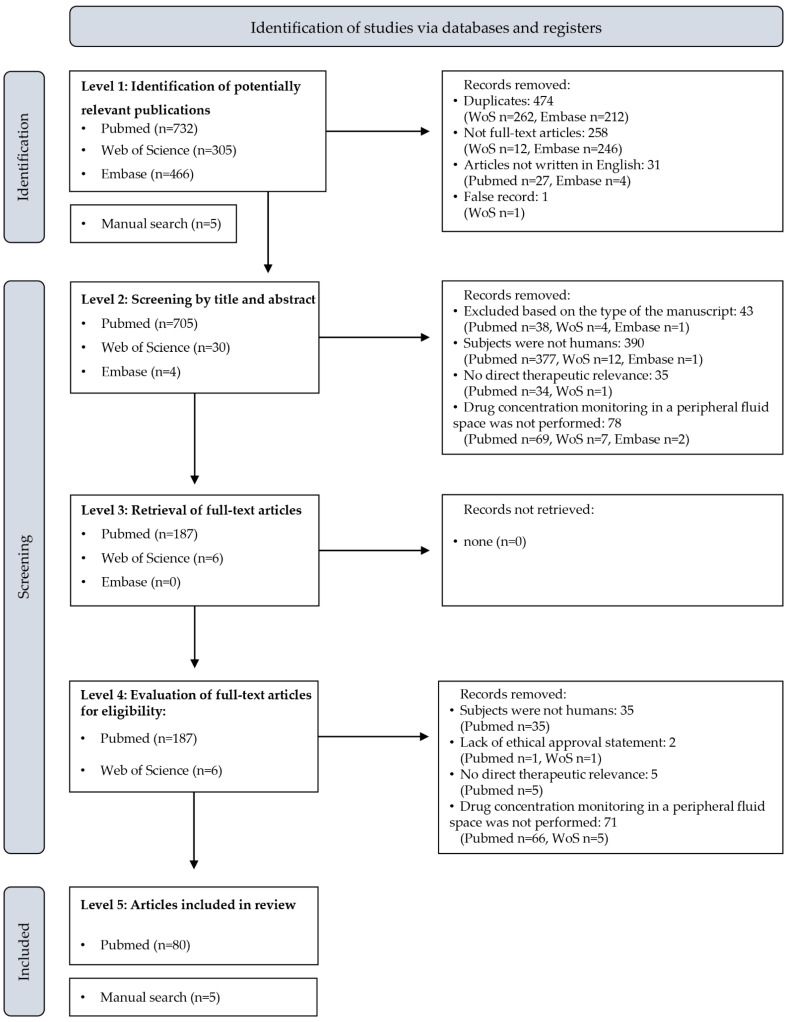
Flowchart of the search strategy and the article selection process. WoS, Web of Science.

The workflow of retrieving research articles for full evaluation is shown in Figure 1. The evaluation of the records was performed by Z.K. and G.B.K.

First, duplicates of the PubMed records were removed from the results of the Embase and Web of Science database search. The remaining records were subsequently assessed individually for meeting basic requirements. Only peer-reviewed full manuscripts written in English, assigned an individual digital object identifier, and made available online by the publisher within the searched period were considered for further screening. Level 2 screening was based on the contents of the title and the abstract. Only records with an explicit evidence of ineligibility were removed at this level. The type of the article was the first object of assessment. Articles presenting randomized and nonrandomized registered clinical studies, non-registered, researcher-initiated clinical studies, retrospective observational studies, case series (describing 2 cases or more with the individual assessment of subjects), and individual case reports were included for further evaluation. Book chapters, comment articles, editorials, meta-analyses, practical guidelines, research protocols, scoping reviews, and systematic reviews were not considered. Second, articles describing experiments in which the subjects were not humans, i.e., in vitro experiments or in vivo animal studies, were removed. Subsequently, studies performed with the participation of human subjects, but without the aim to evaluate or to support decisions related to their medical treatment, i.e., without direct therapeutic relevance (e.g., with the inclusion of healthy volunteers, or conducted with the only aim to deliver pharmacokinetic data), or including medical intervention which, by current understanding, would not be part of the clinical practice (e.g., monitoring drug levels in cord blood or in amniotic fluid to evaluate the exposure of the fetus) were eliminated. Finally, studies explicitly performed without the monitoring of any of the drugs listed in Table 1 in a peripheral fluid space were also excluded.

In the phase of full manuscript screening, the first object of assessment was the ethical review board approval. Case reports and case series were exempt from this requirement. Articles continued to be retained if explicit evidence was found in the main text confirming that the study had been performed with direct therapeutic relevance, as described for level 2 screening. The presentation of the results of monitoring at least one drug displayed in Table 1 in a peripheral fluid space was a further requirement for inclusion. Articles not excluded in this phase were subject to full evaluation. The full manuscripts retrieved by manual search were screened in an identical manner before inclusion.

### 2.3. Data Evaluation and Visualization

All descriptive information on the database records and the contents of the manuscripts found were stored and processed using Microsoft Excel. The year-normalized number of publications on each drug was calculated as n_publ_/n_year_, where n_publ_ is the number of included publications on the drug, and n_year_ is the number of years the drug had been available on the market. The latter was defined as the period starting with the day of the first approval by the American Food and Drug Administration, and ending on 31 August 2022. Visualization was carried out using Microsoft Office applications.

## 3. Results

### 3.1. Summary of the Findings of the Literature Review

The database search yielded a set of 1503 potentially relevant articles (732, 305, and 466 hits in PubMed, Web of Science, and Embase, respectively). Four-hundred and seventy-four duplicates were removed. Of the remaining 1029 papers, 258 were presentation abstracts, and 31 were not written in English. In a single case, a record was listed with false authors. These records were also excluded. The manual search yielded five additional hits which were subsequently found in the PubMed database, but had not been listed by the automatic search. The assessment of the remaining 739 articles based on title and abstract resulted in the exclusion of further 546 publications. Forty-three articles were excluded based on their type. Three hundred and ninety works described in vitro experiments or in vivo animal studies, and 35 were conducted in humans, but without a direct therapeutic goal. Seventy-eight studies were excluded based on evidence retrieved from the title and/or the abstract that drug concentrations were not monitored in any peripheral fluid space.

All of the 193 publications retained for full evaluation could be retrieved from the websites of the publishers. An in-depth study of these manuscripts resulted in the elimination of 113 publications. The authors of two papers failed to present evidence of the approval of an ethical review board for conducting research on humans. Thirty-five studies were not performed in humans, and five were conducted without direct therapeutic relevance. Seventy-one works were excluded because drug concentrations were not monitored in any peripheral fluid space. The remaining 80 publications were selected for the detailed review (Figure 1, Table 2). Overall, 34% of the included publications were individual case reports, 31% were registered clinical studies, 19% were case series, 13% were non-registered, researcher-initiated studies, and 3% were retrospective observational studies.

**Figure 2:**
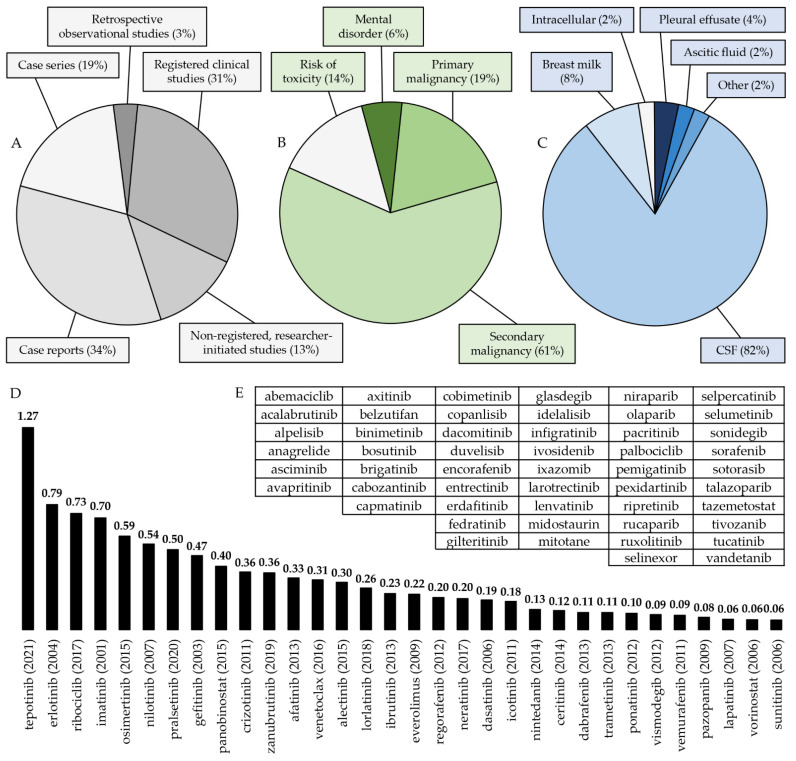
Summary of the results of the literature search. (**A**) Types of studies, (**B**) indications of monitoring, (**C**) monitored peripheral fluid spaces, (**D**) evaluation of clinical interest: number of manuscripts discussing the substance normalized to the number of years on the market, (**E**) marketed small-molecule, orally taken anticancer drugs with cellular protein targets without any example of being monitored in a peripheral fluid space. CSF, cerebrospinal fluid.

Thirty-two small-molecule, orally taken anticancer medications with specific cellular protein targets were monitored with a clinical indication in at least one peripheral liquid space on at least one occasion in the investigated period. This comprises 38.6% of the OACDs which had an ATC code on 31 August 2022. CSF was the most frequently monitored peripheral space (82% of all publications). The share of manuscripts on all other peripheral spaces (breast milk—8%, pleural effusate—4%, ascitic or peritoneal dialysis fluid—2%, intracellular fluid—2%, other (saliva and semen, 1 record for each)—2%) was low. In 61% of the cases, the indication for monitoring an OACD in a peripheral fluid compartment was to control a secondary malignancy. Other indications were the treatment of a primary malignancy (19%), controlling toxicity (14%), and treatment of a mental disorder (6%). In a single manuscript, an additional indication was the prevention of graft rejection [81]. Registered clinical studies, non-registered, researcher-initiated studies, case series, and case reports comprised 29.4%, 14.1%, 22.4%, and 34.1% of the included publications, respectively (Figure 2).

Based on the number of relevant publications available on a specific OACD, normalized to the number of years it has been marketed, tepotinib (Tepmetko^®^) triggered the largest interest, followed by erlotinib (Tarceva^®^), ribociclib (Kisqali^®^), imatinib, and osimertinib (Tagrisso^®^), while the OACDs receiving the least attention were lapatinib (Tyverb^®^), vorinostat (Zolinza^®^) and sunitinib (Sutent^®^, Figure 2, Table 3).

### 3.2. Monitoring the Concentrations of Oral Anticancer Drugs in Peripheral Fluids

#### 3.2.1. Monitoring the Treatment of Primary Malignancies

##### Primary Malignant Central Nervous System Tumors

Currently, the most common indication of monitoring OACDs in a peripheral fluid space is to improve the treatment of primary and secondary CNS tumors in adults and in pediatric patients by performing measurements in the CSF. Early examples for such efforts included the assessment of erlotinib in pediatric glioblastoma and in CNS hemangioblastoma with von Hippel–Lindau disease, and of vismodegib (Erivedge^®^) in pediatric recurrent or refractory medulloblastoma [47,48,94]. Broniscer et al. investigated the pharmacokinetics of erlotinib in a pediatric patient by measuring the concentrations of erlotinib along with its *O*-demethylated, pharmacokinetically active metabolite OSI-420 in plasma and in CSF. Six time-matched pairs of specimens were collected. The CSF/total plasma concentration ratio (CSF-TPR) of erlotinib was 7.0%, while the ratio of drug exposure was 6.9% based on 24-h areas under the concentration-time curves. This evaluation was based on total plasma levels. Since the fraction of erlotinib bound to plasma proteins is approximately 93%, it is reasonable to assume that the unbound fraction equilibrated between plasma and CSF at a 1:1 ratio [47,97]. In a single paired measurement performed in an adult patient, a median erlotinib CSF level corresponding to 21.6% of median total plasma concentrations was found, which would be equivalent to 309% of the unbound plasma fraction [48]. In a phase 1 study conducted with pediatric patients, a total of nine paired CSF and plasma samples were collected from three subjects to evaluate vismodegib concentrations. The CSF/unbound plasma concentration ratios (CSF-UPR) attained a median of 53% (26–78%) [94].

The monitoring of OACDs in this context has gained more attention only very recently. The concentrations of regorafenib (Stivarga^®^) as well as its active *N*-oxide and demethylated *N*-oxide products were assessed in recurrent malignant glioma. All three substances attained detectable levels in CSF. While the concentration values were not explicitly provided by the authors, visual plots showed that the CSF-TPR’s were 0.01 or higher. Approximately 99.5% of circulating regorafenib is bound to proteins, indicating that the CSF levels exceeded unbound plasma concentrations [89].

The monitoring of ceritinib (Zykadia^®^) and ribociclib in patients diagnosed with recurrent glioblastoma was performed [74,78,79]. The unbound fraction of ceritinib, determined using equilibrium dialysis with a 5 kDa regenerated cellulose membrane, corresponded to 1.4% (0.6–2.6%) of total levels. The unbound CSF concentrations were comparable to concentrations measured in nonenhancing tumor regions, and were tenfold higher than unbound plasma levels [74].

The ratio of ribociclib CSF/unbound plasma concentrations was 1.29 in one study and 0.6–4.4 in another. Equilibrium dialysis was employed in both works to determine the unbound fractions directly. The ratios increased over time [78,79]. Ribociclib CSF concentrations were evaluated in recurrent or refractory malignant pediatric brain tumor. The CSF-TPRs were 0.0–42.9% [80].

Dasatinib (Sprycel^®^), imatinib, nintedanib (Ofev^®^), panobinostat (Farydak^®^), regorafenib, ribociclib, and vorinostat were assayed in 42 CSF samples obtained from nine pediatric brain tumor patients. Nintedanib and panobinostat were undetectable in the samples. There was a correlation between blood protein levels and imatinib concentrations. In addition, imatinib and regorafenib proved to bind to CSF proteins as well, resulting in unbound fractions of 88% and 65%, respectively. These data indicate that both plasma and CSF protein concentrations may have an impact on detectable drug levels, and that the elevation of drug availability can be expected in CSF when the blood–brain barrier is not intact and CSF protein levels increase [12].

Ibrutinib (Imbruvica^®^) was measured in CSF in primary CNS lymphomas [85,86]. In one study, hemodialysis was conducted every other day. Six-hour post-dose CSF ibrutinib levels were about tenfold higher on hemodialysis-free days than those observed on hemodialysis days. In addition, the CSF-UPR’s (with an assumed protein-bound fraction comprising 97.3% of circulating ibrutinib) were 78% and 8%, respectively [85].

Zanubrutinib (Brukinsa^®^) concentrations were assayed in 23 time-matched plasma and CSF samples of 13 patients, 8 of whom were diagnosed with primary CNS lymphoma, and 5 with diffuse large B-cell lymphoma. The CSF-TPR was 2.39±1.71%. With an assumed 94% protein binding rate, the authors calculated CSF-UPR’s of 42.7±27.7%, and concluded that zanubrutinib was successfully transported through the blood–brain barrier [87].

Dabrafenib (Tafinlar^®^) and Trametinib (Mekinist^®^) did not reach detectable levels in CSF in patients diagnosed with V600e positive glioma [69].

##### Other Primary Malignancies

Other types of tumors in which OACD concentrations have been evaluated in peripheral fluid spaces include Philadelphia chromosome-positive (Ph + ) chronic myeloid leukemia (CML), non-small cell lung cancer, and gastrointestinal stromal tumors.

Imatinib concentrations were monitored in patients diagnosed with Ph + CML. In a follow-up study conducted with 15 adult patients, Nambu et al. found a weak correlation between imatinib levels determined in leukocytes (buffy coat cells) and in plasma (r = 0.281). While the intracellular concentrations of the drug were not associated with the cytogenic response, there was a significant difference between groups of patients with different genotypes (SLCO1B3 334TT and 334 TG/GG) [13]. In another study conducted with adult Ph + CML subjects, peripheral blood mononuclear cells (PBMC) were isolated from anticoagulated whole blood. Again, a weak yet statistically significant positive correlation was found between imatinib concentrations observed in plasma and in PBMC (r = 0.203) [14]. In both works, intracellular imatinib concentrations were about a magnitude higher than those found in plasma.

Malignant pleural effusion is a severe condition developing as a complication of lung or breast cancer in women [98]. Masago et al. investigated erlotinib and OSI-420 concentrations in the plasma and in the pleural effusate samples of nine adult patients diagnosed with advanced NSCLC. On days 1 and 8 of the treatment, 2-h post-dose (day 1) and trough pleural effusate levels (day 8) were compared to trough plasma concentrations. They found that erlotinib and OSI-420 pleural effusate concentrations had increased considerably, with larger than 100% pleural effusate/total plasma concentration ratios obtained by day 8 [49]. In an NSCLC patient, gefitinib concentrations in pleural effusates attained approximately 30% of those observed in plasma. The penetration of gefitinib into the peritoneal third-space fluid was, on the other hand, negligible [38].

#### 3.2.2. Monitoring the Treatment of Malignant Tumor Metastases

##### Central Nervous System Metastases of Myeloproliferative Malignancies

The involvement of the CNS presents a major challenge in the therapy of leukemias. Adult patients present with CNS leukemia in approximately 5% of acute leukemia cases, while CNS involvement occurs in about every third pediatric patient presenting with a relapse [99]. The risk of malignant cell penetration through the blood–brain barrier is especially high in Ph + B-cell precursor acute lymphoid leukemias (ALL) [100]. The prevention of CNS involvement in acute leukemias and the efficient treatment of established CNS leukemias are, therefore, of considerable importance and have an impact on the overall survival.

The poor penetration of the blood–brain barrier by imatinib, the first marketed tyrosine kinase inhibitor drug, was first mentioned in 2002 [15,16]. The total imatinib concentrations were 1.57 µg/mL and 0.017 µg/mL in the plasma and CSF samples of a young female adult diagnosed with Ph + ALL [16]. The size of the unbound fraction of imatinib was later established to be around 5% (4.3–6.5%) in healthy humans and in acute myeloid leukemia patients. By applying this percentage, the CSF-UPR of imatinib in this patient can be estimated as 21.7%. While the authors concluded that the distribution of imatinib into CSF was extremely poor, the consideration of the unbound fraction as the basis of the evaluation of blood–brain-barrier penetration delivers a more appreciable penetration rate [101].

On five separate days in an 11-day period, measurable imatinib concentrations were found in the CSF and plasma samples of a male Ph + CML patient who was in a lymphoid blast crisis after achieving complete cytogenic remission in the bone marrow following more than eight months of imatinib therapy, but had developed an isolated neoplastic meningitis. The authors concluded that imatinib CSF concentrations were not sufficient to inhibit 50% of BCR/ABL tyrosine kinase, and assume that the reason underlying the poor penetration of imatinib is its affinity to p-glycoprotein, a protein responsible for multi-drug resistance. Nevertheless, total imatinib concentrations were evaluated, and by calculating its unbound plasma concentrations, imatinib CSF-UPR’s can be established as 7.7–56.2% [15]. Further investigations confirmed these findings. Imatinib CSF-TPR was 2.6% in a patient diagnosed with a CSF lymphoid blast crisis, while displaying a major cytogenic response in the bone marrow after 16 months of imatinib treatment. This corresponds to a calculated CSF-UPR of 52.6% [17]. In a randomized, multicenter phase 2 trial, plasma and CSF samples were collected from 17 BCR/ABL + ALL subjects with or without meningeosis and receiving imatinib. The CSF-TPRs were 1.8%. [18]. Imatinib CSF and plasma concentrations were further evaluated in parallel in four adult subjects of a multicenter clinical trial. One of the subjects was a biphenotypic Ph + CML patient, while the other three had been diagnosed with Ph + ALL. The CSF concentrations (mean: 0.044 µg/mL) were 74-fold lower than total plasma concentrations (3.27 µg/mL), corresponding to a CSF-UPR of 26.9% [19].

Dasatinib concentrations were below the detection limit in the CSF of a female adult patient treated with Ph + ALL and an extramedullary and meningeal relapse following bone marrow transplantation. The trough plasma dasatinib concentration was 32 ng/mL (CSF-TPR: 0.23–1.5%) [27]. Following the detection of large individual variability in the systemic exposure to dasatinib, Gong et al. measured pairs of the CSF and plasma concentrations of the substance in five Ph + ALL adult patients after giving doses of 100 mg or 140 mg. Only two pairs of samples contained dasatinib in quantifiable concentrations in both media. The CSF-TPRs were 0.75% and 1.42%, while the calculated CSF-UPRs were 18.7% and 37.2% [28].

Four leukemia patients (three diagnosed with Ph + ALL and one with Ph + CML and a blast crisis), all with CNS relapse after allogeneic stem cell transplantation, received nilotinib (Tasigna^®^). Seventeen matched pairs of CSF and plasma samples were collected. The CSF-UPRs were calculated by taking a 98% protein binding rate into account. The calculated concentration ratios were 12%, 20%, 30%, and 68%, pointing to large individual differences in the availability of the drug [29]. In a group comprising 30 Ph + ALL patients aged 15 years or older, only non-quantifiable traces of nilotinib were found in the CSF samples collected [30].

The penetration of the selective BCL2-inhibitor venetoclax (Venclyxto^®^) through the blood–brain barrier was also poor; however, it corresponded to the in vitro IC_50_ of the drug in an adult, male chronic lymphocytic leukemia patient diagnosed with trisomy 12, IGHV unmutated (VH4L) chronic lymphoid leukemia and experiencing a CNS relapse. Time-matched pairs of plasma and CSF samples were assayed after their collection in steady state, after 2 h and 23 h of drug intake, with 0.23% and 2.89% concentration ratios obtained. The unbound fraction of venetoclax is smaller than 1% of the total circulating amount; therefore, the CSF concentrations corresponded to approximately 10–29% of the unbound plasma levels [95]. Venetoclax concentrations were evaluated 23, 30, and 37 days after initiating treatment in another male adult patient presenting with a complete remission in the bone marrow after hematopoietic stem cell transplant, but with a blast crisis detected in the CNS, and formerly receiving other chemotherapy. The CSF-TPRs were 0.32–0.40%, corresponding to CSF-UPRs of at least 32–40% [96].

An extremely low CSF concentration (0.1 ng/mL) of ponatinib (Iclusig^®^), another very heavily ( > 99%) protein-bound drug, was observed in a 3-year old girl diagnosed with Ph + acute lymphoblastic leukemia which had been confirmed to have penetrated the CNS [37].

##### Central Nervous System Metastases of Non Small-Cell Lung Cancer

**Figure 3:**
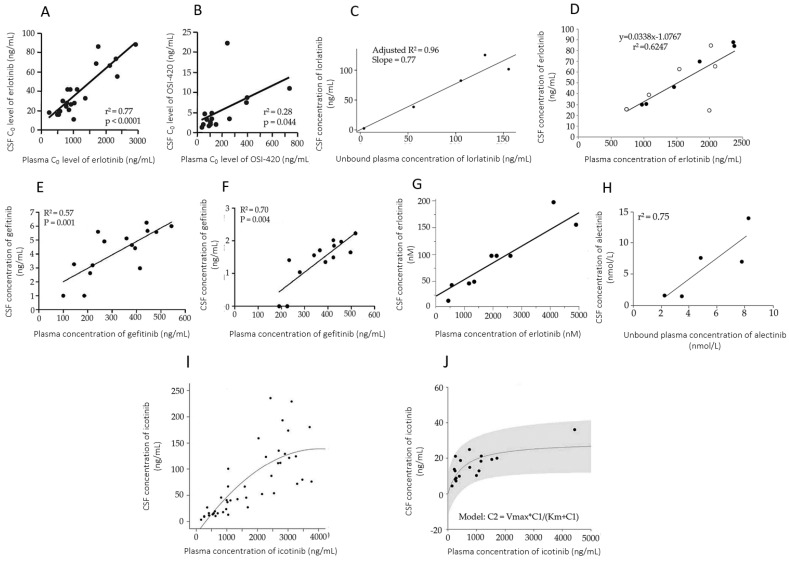
Relationships of the concentrations of various orally administered, small-molecule anticancer drugs with specific cellular protein targets in serum/plasma and in cerebrospinal fluid (CSF). (**A**) Erlotinib in plasma and in CSF, trough samples were drawn. (**B**) O-desmethyl-erlotinib (OSI-420) in plasma and in CSF, trough samples were drawn. (**C**) Unbound alectinib in plasma and in CSF. (**D**,**E**) Gefitinib in plasma and in CSF. (**F**) Unbound lorlatinib in plasma and in CSF. (**G**,**H**) Erlotinib in plasma and in CSF. (**I**) Gefitinib in plasma and in CSF, a Michaelis–Menten equation has been fitted to the data. (**J**) Icotinib in plasma and in CSF [50,61,62,63,64,70,77,83]. Licenses or permissions to reproduce the graphs have been granted by the copyright holders.

The first manuscript discussing the quantitation of OACDs in CSF for monitoring their efficacy regarding the treatment of the CNS metastases of NSCLC was published on the epidermal growth factor receptor inhibitor gefitinib (Iressa^®^). This was a case report presenting a Japanese male patient diagnosed with NSCLC and developing carcinomatous meningitis. Ten days after the initiation of gefitinib treatment, the drug was assayed in serum and in CSF before and 2 h after the intake of 250 mg drug. At both time points, the observed CSF concentrations were negligible, 0.9 nmol/L, while serum concentrations of 117 and 132 nmol/L were attained. Assuming a 97% protein binding rate, this corresponds to CSF/unbound serum concentration ratios of 22.7% and 25.6% [39,102]. Interestingly, significant positive linear correlations of gefitinib CSF and plasma levels were revealed in multiple research works (Figure 3) [40,41,42]. In contrast, the results of a phase 1 open-label trial of a novel, high-dose gefitinib treatment conducted with the involvement of seven patients diagnosed with leptomeningeal metastases of NSCLC showed that this approach did not result in an improved penetration of gefitinib into the CSF [43]. Evidence exists for supporting that the low penetration rate of gefitinib may be increased by whole-brain radiotherapy, an intervention considered to be an efficient strategy to improve blood–brain barrier permeability [41]. However, contrasting results have also been published [44]. A direct comparison of the concentrations of gefitinib and erlotinib, which have similar chemical structures, in the CSF of patients diagnosed with leptomeningeal metastases, resulted in the conclusion that erlotinib attained higher molar concentrations and a higher rate of penetration into the CNS [45].

Two years after the publication of the first measurement of gefitinib concentrations in CSF, erlotinib concentrations were evaluated in three lung adenocarcinoma patients developing leptomeningeal metastases during gefitinib therapy. Twenty-eight days after switching to erlotinib, clinical improvement was observed, accompanied by 2.5–13.3% CSF-TPRs, corresponding to CSF-UPRs of 36–190% [50,97]. Four cases of Asian female adult NSCLC patients who had developed adenocarcinoma as a CNS metastasis and started to receive 150 mg erlotinib once daily were described by Togashi et al. Matched pairs of CSF and plasma samples were collected on day 8 of the treatment. Similar penetration of the drug and its active metabolite OSI-420 into the CSF was found. The authors provided the CSF-TPRs and the CSF concentrations, which allows the calculation of total and unbound plasma concentrations, as well as CSF-UPRs (45.7–110%). The efficiency of erlotinib to penetrate the blood–brain barrier was concluded to be higher than that of gefitinib, and allows the effective treatment of EGFR wild-type cases as well [51]. Yet another study involving six adult NSCLC patients with brain metastases confirmed that erlotinib could reach a mean penetration rate of 4.4%, corresponding to a CSF-UPR of 47.2%. The CSF concentrations of the drug were associated with the outcome, with the highest levels attained in patients showing partial response to therapy, and the lowest seen in those with progression [52]. At steady state, the CSF penetration rate of erlotinib was determined as 5.6% (corresponding to a CSF-UPR of 77.0%) in a female patient diagnosed with stage IV lung cancer and stage I breast cancer, and receiving a combination of erlotinib and bevacizumab [53]. A considerably lower ratio of 1.15% (corresponding to a CSF-UPR of 16.4%) was observed, however, in a woman with stage IV NSCLC and leptomeningeal metastasis and receiving 1500 mg erlotinib weekly [54]. A similarly low penetration rate of erlotinib (1.6–2.6%) was identified in six Chinese adult NSCLC patients with leptomeningeal metastasis refractory to gefitinib treatment. Three patients received premetrexed and cisplatin in addition to erlotinib, while the other three received only erlotinib. There was no difference in the penetration rates between the two patient groups. The calculated CSF-UPRs were 22.8–36.6% [55]. A very strong linear correlation was identified, at the same time, between plasma and CSF erlotinib concentrations [56]. This finding was also confirmed by another study (Figure 3) [57]. A phase 2 single arm trial was conducted to reveal the efficacy of erlotinib in stage IV NSCLC with leptomeningeal metastasis (LOGIK11001) by Nosaki et al. The primary endpoint was the cytological clearance rate, and the secondary endpoints were time to disease progression, overall survival, toxicity, and quality of life. Plasma and CSF concentrations of erlotinib were determined in single steady-state samples collected from 12 participants. The mean penetration rate was 2.9–12.1%, corresponding to CSF/unbound concentration ratios of 41.9–173%. Again, a good correlation was observed between the plasma and the CSF concentrations (R^2^ = 0.6247), regardless of the cytological response Figure 3 [58].

In a comparative study conducted to evaluate the penetration rate of standard (150 mg/die and 250 mg/die, respectively, administered for seven days) versus pulsatile high-dose erlotinib (1500 mg on day eight and fifteen) and gefitinib (2500 mg/die from day eight to fifteen) in NSCLC patients with brain metastases who progressed on standard doses, both drugs attained higher concentrations in the CSF as a result of high-dose administration, with a constant CSF-TPR of 2% in the case of erlotinib, and a saturable penetration rate of gefitinib with no increases in CSF levels predicted for doses of 839 mg or higher (Figure 3). In addition, those undergoing whole-brain radiotherapy attained disproportionately higher CSF concentrations of the drugs. Adverse effects were more prevalent in patients receiving erlotinib, with the high doses of gefitinib being well tolerated [46].

The next drug assayed in the CSF was crizotinib (Xalkori^®^), with negligible blood–brain barrier penetration rates observed. A CSF-TPR of 0.26% (corresponding to a CSF-UPR of 2.89%, assuming a 9% unbound fraction of the drug) was found in a 29-year old Caucasian male diagnosed with stage IV NSCLC and treated first with cisplatin plus pemetrexed, then with erlotinib, and finally with crizotinib. The attained CSF concentration was substantially lower than the established 50% inhibitory concentration (IC_50_) required to inhibit mutant cell lines against which crizotinib had been tested [70,103]. In two ALK-positive male adult NSCLC patients developing brain metastases, crizotinib CSF/total serum concentration ratio was 0.06% and 0.1%, corresponding to CSF/unbound serum concentration ratios of 0.66% and 1.1%, respectively [71]. Three CSF samples of a 60-year-old male patient diagnosed with ALK-rearrangement-positive NSCLC and receiving 250 mg crizotinib twice daily after developing brain metastases were assayed for crizotinib at one-week intervals following whole brain radiotherapy (an additional sample was processed before conducting WBRT). Crizotinib was undetectable in the samples collected before and one week after WBRT, while 6.2 and 6.3 ng/mL concentrations were found after two and three weeks, respectively, accounting for 3.5% and 2.2% of the total, and for 39.0% and 24.5% unbound plasma concentrations [72]. In another male patient diagnosed with stage IIA lung adenocarcinoma and brain metastasis, a CSF-TPR of 2.6% (corresponding to a CSF-UPR of 30.4%) was achieved at a single sampling point following WBRT. The CNS symptoms diminished, and the negativity of CSF to malignant cells was confirmed. Comparing this result to earlier findings yielded the conclusion that WBRT may enhance the CNS penetration and the clinical efficacy of crizotinib [73].

In a single-arm, open-label, multicenter phase 1/2 study conducted with the involvement of adult subjects with histologically confirmed, locally advanced or metastatic NSCLC with crizotinib-resistant ALK-positive rearrangement and receiving 600 mg or 900 mg alectinib (Alecensa^®^) twice a day in the fixed dose phase, five matched alectinib CSF-plasma concentration pairs were obtained. The CSF concentrations not only showed positive correlation with the unbound plasma fraction of alectinib (which corresponded to 0.3% of the total amount), but were also equivalent or higher. The extrapolated trough CSF concentration exceeded the reported in vitro IC_50_ of alectinib for ALK inhibition [75]. In an institutional case series comprising eleven adult subjects diagnosed with histologically confirmed ALK-positive NSCLC and receiving 600 mg alectinib twice daily until disease progression, unacceptable toxicity or withdrawal of consent, matched CSF-serum concentration pairs were obtained in two patients in the second month of alectinib therapy. The total serum concentrations were 694 ng/mL and 707 ng/mL, both corresponding to 2.1 ng/mL unbound serum concentrations. The calculated CSF/unbound serum concentration ratios were, therefore, 100% and 30% in the two patients [76].

The evaluation of afatinib (Giotrif^®^) CSF levels was first described in a woman diagnosed with stage IV adenocarcinoma of the lung with an underlying mutation of the EGFR gene. Two CSF samples were assayed, and afatinib was found to attain a penetration rate lower than 1%, with a calculated CSF-UPR of 13.9% when 95% protein binding rate of the drug is assumed [59,104]. A remarkable case of a female patient diagnosed ten years earlier reporting with stage IV adenocarcinoma of the lung with an EGFR mutation was also described. Afatinib (40 mg/die, deescalated to 30 mg/die after four months) was administered as the eighth line of treatment following interchanging periods of progression and remission. Trough plasma and CSF concentrations were assayed at three, four and five months following the initiation of afatinib dosing. The CSF-TPR’s were 0.28–0.40%, while the calculated CSF-UPRs are 7.5–8.8%. The total plasma concentrations were 19.0–33.4 ng/mL, which can be measured with relative convenience using liquid chromatography–tandem mass spectrometry (LC–MS/MS), but the obtained CSF levels of 0.05–0.14 ng/mL clearly indicate that assaying afatinib in the CSF is a major analytical challenge [60]. Further, a prospective multicenter trial was conducted with the involvement of 11 patients diagnosed with EGFR mutation-positive NSCLC with leptomeningeal carcinomatosis and with the aim of evaluating the CSF penetration rates and the clinical efficacy of afatinib. Participants received 40 mg afatinib once a day. On day eight, the trough concentrations were assayed in plasma and in CSF. Afatinib could be quantitated in the CSF samples of eight subjects (72.7%). The CSF-TPRs were 0.1–3.1%, with a single case of 9.3% which resulted from an unusually low plasma concentration (corresponding to 44.4% of the next value in the ranked series of the measured concentrations), accompanied by the second-highest CSF concentration. This corresponds to CSF-UPRs of 2.1–185%. It was concluded that the ability of EGFR tyrosine kinase inhibitors to penetrate the CSF should be assessed along with the efficacy of the drug against tumors with particular mutation types [61].

The penetration of icotinib (Conmana^®^), an OACD currently approved in China, into CSF was first evaluated in a phase 2 clinical study involving ten patients following the administration of 125 mg in a three-times-per-day regime. Meanwhile, WBRT was delivered in 3-Gy fractions once per day, five days per week, to a total dose of 30 Gy. The mean total plasma concentrations were 940.6±503.8 ng/mL (corresponding to 47.0±25.2 ng/mL unbound concentrations), while the mean CSF concentrations were 11.6±9.1 ng/mL in samples collected two hours after drug intake. The CSF-TPR was 1.4±1.1%, and the mean CSF-UPR can be calculated as 24.7% [66]. The impact of WBRT on the CSF penetration of icotinib was directly investigated in fifteen patients receiving escalating dose levels (125–352 mg) three times a day. Blood and CSF samples were collected immediately before beginning the WBRT treatment (applied in fixed doses of 37.5 Gy, five times a week, lasting for three weeks), immediately after terminating WBRT therapy, and four weeks into the follow-up period. The CSF-TPR’s of icotinib were 2.4–3.7% in a dose range of 125–500 mg (peculiarly, 6.1% at 375 mg), while the CSF-UPR can be calculated as 52.0–58.0% (130% at 375 mg) [67].

The CSF concentrations of osimertinib were first measured in an NSCLC patient with leptomeningeal metastases and EGFR-TKI resistance. A poor penetration rate (1.47%) was observed [62]. In an open-label, single-arm, multicenter, prospective study (APOLLO), twelve adult patients donated matched blood and CSF samples. The evaluation of osimertinib concentrations was based on the unbound drug fractions. A strong linear correlation was found between blood and CSF levels (r = 0.8306). Based on these calculations, the median CSF-UPR of osimertinib was 31.7% (19.8–57.8%) after six weeks of treatment [63]. In a phase 2 study involving radiotherapy-naive adult patients diagnosed with T790M EGFR mutation-positive NSCLC and CNS metastasis, who had been previously treated with EGFR tyrosine kinase inhibitors, the plasma and CSF concentrations of osimertinib and its pharmacologically active metabolite were assessed in seven participants on day twenty-two of osimertinib therapy. The CSF-TPRs of the drug and the metabolite were 0.79% (0.43–1.32%) and 0.53% (0.31–0.64%), respectively, corresponding to 15.8% (8.6–26.4%) in the case of the parent drug by assuming 99% plasma protein binding rate [64].

Tepotinib plasma and CSF concentrations were evaluated in a male adult patient diagnosed with stage IIIA lung adenocarcinoma. EGFR mutation and ALK fusion gene were not detected. Following right lung pneumonectomy, a brain metastasis was identified in the left cerebrum which later progressed to leptomeningeal metastasis and hydrocephalus in spite of treatment with cisplatin and pemetrexed. A tepotinib regimen (500 mg/die) was started. On day 20 of therapy, the tepotinib CSF-TPR achieved 1.83% in the matched samples collected four hours post-dose. The attained concentration was judged to have exceeded the IC_50_ [90]. In a female patient diagnosed with NSCLC with MET exon 14 skipping mutation and with brain metastases, and having received WBRT, remarkable clinical improvement was achieved after a 1-month treatment with tepotinib (500 mg/die). The penetration rates of tepotinib into the CSF at two, four and eight weeks of therapy were 1.19%, 1.42%, and 1.73%, respectively. By taking the 98% protein binding rate of tepotinib into account, the CSF-UPRs can be calculated as 60.0%, 71.1%, and 86.6%, respectively, based on the data described by the authors [91].

Lorlatinib (Lorviqua^®^) was monitored in the CSF in an ongoing, open-arm, multicenter phase 1/2 trial with the aim to further investigate the penetration of the drug into the CNS. Five patients with suspected or confirmed leptomeningeal carcinomatosis not visualized on magnetic resonance imaging, or carcinomatous meningitis, were included. Samples were collected at baseline and a later yet undefined point of the study. The CSF/plasma unbound lorlatinib concentration ratios were 61–96%, and showed very strong correlation (adjusted r^2^ = 0.96). The CSF/total plasma lorlatinib concentration ratios were 21–33%. The results indicated that lorlatinib concentrations exceeded the minimum efficacy concentrations in all of the patients regarding wild-type anaplastic lymphoma kinase (ALK) and the L1196M ALK resistance mutation. The authors concluded that this supported the broad coverage of these mutations, and, in approximately one-third of patients, the coverage of the G1202R ALK resistance mutation [77].

The CSF concentrations of pralsetinib (Gavreto^®^) and osimertinib were investigated in an adult patient with an EGFR-mutant NSCLC with acquired RET fusions and meningeal metastasis after four months of co-treatment with pralsetinib and osimertinib. Pralsetinib attained concentrations of 91.3 µmol/L and 0.705 µmol/L in plasma and CSF, respectively (ratio: 0.77%, corresponding to a CSF-UPR of 15.4%). Osimertinib concentrations were 2.149 µmol/l and 0.0237 µmol/L, respectively (ratio: 1.10%, corresponding to a CSF-UPR of 110%). Despite the lower CSF/unbound concentration ratios, pralsetinib levels were judged to be sufficiently high both in plasma and in CSF to inhibit the CCDC6-RET-mutated protein, indicating that pralsetinib is more efficient than osimertinib to treat this mutation [65].

##### Metastases of Other Malignancies in the Central Nervous System

Lapatinib inhibits both EGFR and HER2; therefore, it has activity against brain metastases developing from HER2-positive metastatic breast cancer. This activity may be enhanced by combining lapatinib with capecitabine. Nevertheless, 0.9–1.3% of CSF-TPRs of lapatinib were observed in two adult female patients diagnosed with HER2-positive (one HR-negative and one HR-positive) ductal carcinoma yielding CNS metastases. The CSF-UPRs can be calculated as 8.6–12.9% in these two patients [83,105]. Neratinib, another HER2 tyrosine kinase inhibitor, was absent (<1.50 ng/mL) in the CSF samples of three adult HER2-positive breast cancer patients [84]. Vemurafenib was, on the other hand, quantitated successfully in the matched CSF and plasma samples of patients treated with the drug in a dose of 960 mg, given twice daily, for brain metastatic BRAF-V600 mutated melanoma. The CSF-UPRs were 28–250%, assuming a 99% protein binding rate [68,106].

##### Malignant Ascites

Malignant ascites is a rare condition secondary to abdominal malignancies [107]. In an elderly adult patient diagnosed with papillary renal cell carcinoma and undergoing treatment first with pazopanib (Votrient^®^), then with sunitinib, concentrations of the administered OACD were monitored in plasma and in ascitic fluid. The concentrations measured in the ascitic fluid were equivalent to or higher than those assayed in the systemic circulation, and, following an early phase with sufficient plasma levels, systemic concentrations became subtherapeutic [88]. The ascited fluid concentrations of the drugs remained high after discontinuation of treatment. While the underlying reason of the accumulation of these drugs in the ascitic fluid is not evident, it was proposed that it acted as a sink of the administered OACDs, while the strong binding of pazopanib and sunitinib to albumin may have facilitated the extravasation of the drugs.

#### 3.2.3. Monitoring OACDs to Control Toxicity

##### Monitoring the Exposure of the Infant to the Drug during Breastfeeding

CML occurs very rarely during pregnancy, at an estimated rate of 1:750 000. Imatinib is employed for treating Ph + cases developing during pregnancy, an approach which may cause harm to the fetus and the newborn. Assaying the drug in breast milk is valuable for characterizing the exposure of the infant. The first appearance of the measurement of imatinib in breast milk was the description of a case with the imatinib concentrations being approximately 60% of the lower limit of the currently accepted blood reference range (1000–3000 ng/mL). Its pharmacologically active metabolite, however, displayed accumulation in breast milk [20]. Another patient on 400 mg once-daily imatinib donated blood and breast milk samples on a single day, 1, 2, 3, 4, and 9 h after drug intake. The concentrations of imatinib and its active metabolite in milk reached 0.5 and 0.9 of those found in plasma, respectively. The authors concluded that the maximum intake of the infant was 3 mg imatinib/day, and should be considered safe [21]. A case described two years later described a Ph + CML patient receiving the same dose resulting in complete hematological and cytogenetic remission. Blood was drawn on day 2, while breast milk was collected on days 7, 14, 15, and 16 postpartum. The imatinib concentrations measured both in plasma (2385 ng/mL) and in breast milk (1430–2623 ng/mL) were in the therapeutic range. The authors concluded that, since the long-term effects of imatinib on infants are unknown, breastfeeding is not advisable when imatinib is administered [22]. This conclusion was confirmed by the presenters of another case when imatinib treatment was initiated immediately after delivery. While the concentrations of imatinib were relatively low in breast milk, those of the active metabolite attained threefold concentrations of those measured in plasma, clearly displaying accumulation [23]. Yet in another patient, the concentrations of imatinib and the active metabolite were measured in breast milk 99 h after the last intake. The attained concentrations were 19 ng/mL and 600 ng/mL, respectively, pointing to a very significant accumulation of the metabolite in breast milk. Neonatal urine was also evaluated, with 90 ng/mL imatinib and 165 ng/mL active metabolite concentrations detected. These results indicate that the infant was exposed to the drug, and, to an even greater extent, to the metabolite. This case raises the clinical relevance of assessing the concentrations of oral anticancer medications taken during pregnancy in neonatal urine for evaluating the potential impacts on the newborns [24]. In the most recently published case report the milk/plasma ratio of imatinib attained 0.35 at 5 days postpartum. Blood was also collected from the infant on the same day to reveal a 27-ng/mL concentration of imatinib, which was considered to be safe by the authors [25].

Everolimus is primarily administered as an immunosuppressant based on its ability to inhibit the mammalian target of rapamycin (mTOR) functional complex mTORC1. In a heart-transplanted patient, everolimus therapy was continued during pregnancy and following delivery. At 48 h postpartum, the drug was not detectable in the colostrum, indicating that the evaluation of the immunosuppression of the newborn had to be based on its prepartum administration [81].

##### Monitoring Other Types of Toxicity

Oral anticancer medications have serious adverse effects, including low blood cell counts, resulting in an increased susceptibility to infections and, potentially, bleeding, as well as dermal and gastrointestinal symptoms. Several of these may prompt the discontinuation of therapy. Efforts have therefore been made to identify the relationships between the presentation of the drugs in non-targeted organs and fluid compartments, and the development of adverse symptoms.

Pleural effusion may be induced by tyrosine kinase inhibitors. In a young male adult patient who had developed pleural effusion from dasatinib earlier, nilotinib therapy again led to the formation of the effusate. The measured nilotinib concentrations were 927 ng/mL and 2092 ng/mL in plasma and in the pleural effusate, respectively, clearly indicating the accumulation of nilotinib in the latter medium. Other possible causes, including malignancy, were excluded. The severity of this adverse effect is shown by the fact that, eventually, performing endotracheal intubation and left thoracic drainage was required [31].

The relationship between the occurrence of stomatitis and everolimus (Afinitor^®^) levels in saliva was investigated in 11 cancer patients receiving everolimus in a once-daily (10 mg) or twice-daily (2 × 5 mg) regime. Both the plasma and saliva concentrations of the drug were higher in patients with stomatitis than in those who did not develop this condition. While the statistical significance of this difference was low, this result may indicate the utility of everolimus saliva assays concerning the prevention of the occurrence of stomatitis. Of note, the rate of the penetration of everolimus into saliva was extremely low (0.8%) with high interindividual variability (67.7%) [82].

Imatinib has been demonstrated to cross the brain–testis barrier and to reach equilibrium. Imatinib concentrations reached concentrations of 1471 ± 570 ng/mL and 1397±425 ng/mL in the plasma and the semen of eleven male CML patients, respectively. The clinical relevance of the assay was confirmed by the finding that the number, the survival rate, and the activity of sperms were reduced in these patients. Reproductive hormone structures and sex hormone concentrations were unaffected [26].

Panobinostat was not detected in the CSF of patients diagnosed with human immunodeficiency virus (HIV) infection [92]. In addition, it was not present in the CSF samples of pediatric patients with refractory hematological malignancies [93]. It was concluded in these works that panobinostat did not cause CNS symptoms.

#### 3.2.4. Monitoring the Treatment of Mental Disorders

It is increasingly acknowledged that certain OACDs may be effective against neurodegenerative and autoimmune diseases [9,108]. Nilotinib, a BCR-ABL tyrosine kinase inhibitor, has been investigated in multiple cases as a medication against mental disorders, such as Parkinson’s disease and Alzheimer’s disease [109,110]. The rationale of these indications is that nilotinib leads to the degradation of misfolded α-synuclein by autophagy [111]. In addition, in preclinical studies, nilotinib increased dopaminergic neuron survival in the CNS, and improved motor and cognitive outcomes in in vivo models. Abl inhibition has been demonstrated to reduce oxidative stress, and to protect dopaminergic neurons [112].

In an open label pilot study conducted to investigate the safety and tolerability of two doses of nilotinib, the drug penetrated readily into the CSF, and remained detectable there for five hours, when administered to stage 3–5 Parkinson’s disease patients in low doses (150–300 mg/die). This was accompanied by a steady increase in plasma concentrations. The CSF-TPR was higher when the lower dose was administered, with a comparable level of Abl inhibition [32]. Further research revealed that the penetration of nilotinib into CSF was dose-dependent in the dose range 150–400 mg, with 200 mg exerting optimal effects. Again, the CSF-TPR was very similar at various doses (0.5–1.0%). Nevertheless, avoiding higher doses was recommended since more side- and off-target effects were detected in the CNS [33]. A phase 2 randomized clinical trial was published with the involvement of 75 participants, 50 of whom received nilotinib. The CSF-TPR was considerably lower, 0.33% and 0.53% after applying 150 mg and 300 mg nilotinib, respectively [34].

The most recent evaluation of nilotinib delivered results which contradicted some key findings of the above works, although the safety and tolerability of low-dose nilotinib was still acceptable. The measured CSF penetration was in concert with previous findings. This was a six-month, multicenter, randomized, parallel-group, double-blind, placebo-controlled trial conducted with the involvement of 76 participants, 51 of whom received nilotinib (150 mg or 300 mg pro die). Based on the evaluation of the geometric means of measured drug concentrations, the administration of 150 mg or 300 mg resulted in 0.61–1.10 ng/mL and 1.10–1.90 ng/mL peak CSF nilotinib concentrations along with 343.3–524.4 ng/mL and 485.8–621.2 ng/mL peak serum concentrations, respectively, after three months of treatment. This corresponded to 0.16–0.23% and 0.20–0.32% CSF penetration rates, respectively. In contrast to the favorable outcomes of the earlier studies, this trial ended with the conclusion that the low penetration rates were associated with no treatment-related alterations of dopamine metabolites in the CSF. Therefore, the changes in the protein biomarkers (α-synuclein, phospho-α-synuclein, and phospho-tau) alone provided weak evidence of the clinical efficacy of nilotinib treatment [35].

In animal models of neurodegeneration, nilotinib promoted the degradation of proteins Aβ/amyloid protein and the microtubule-associated protein tau [113]. This result prompted a phase 2, randomized, double-blind, placebo-controlled study to evaluate the effects of nilotinib in mild to moderate Alzheimer’s disease. The label arm received 150 mg nilotinib daily for 6 months, followed by 300 mg daily for another 6 months. The ratios of the mean CSF and plasma concentrations were 0.29% and 0.27% at 150 mg and 300 mg doses, respectively. The ratios of the areas under the concentration-time curves were 0.30% and 0.33%, respectively [36].

The above works included the detailed evaluation of quantitative changes in the pharmacodynamic variables, such as microtubule-associated protein tau or amyloid proteins. Although the penetration rate of nilotinib into the CSF was very low, these were associated with statistically significant pharmacodynamic improvement and measurable clinical efficacy.

### 3.3. Bioanalytical Methods of Monitoring OACD Concentrations in Peripheral Fluid Spaces

All of the described bioanalytical methods relied on chromatographic separation using high-performance or ultra-high performance liquid chromatography. Mass spectrometry was chosen for detection by most authors, but multiple examples of applying ultraviolet–visible (UV–VIS) light absorbance detection for the assessment of afatinib, erlotinib, gefitinib, imatinib, nilotinib, and vemurafenib were found.

In the majority of cases, sample pretreatment consisted of deproteinization. The removal of proteins was performed using organic solvents (acetonitrile—ceritinib [74], erlotinib [47], gefitinib [45], ibrutinib [85,86], imatinib [16,20,21,22], ponatinib [37], regorafenib [89], ribociclib [78,79], vemurafenib [68], venetoclax [95], zanubrutinib [86]; acetonitrile-methanol 1:1—dasatinib [27], imatinib [14], nilotinib [32,33,34,36]; acetonitrile-methanol 1:4—erlotinib [54]; acetonitrile-methanol 10:1—zanubrutinib [87]; methanol—alectinib [76], dabrafenib [69], dasatinib [12], erlotinib [46], nintedanib [12], panobinosat [12], regorafenib [12], ribociclib [12], trametinib [69], vorinostat [12]), and, in a single case, the aqueous dilution of perchloric acid (imatinib [18]). The deproteinization methodology was not described by Xing et al. for the monitoring of osimertinib in CSF [63]. PBMC pellets and breast milk were pretreated with acetonitrile-methanol [14] and acetonitrile [20,21,22], respectively, as part of the employed imatinib assays. In all other cases, deproteinization was applied to CSF samples.

Liquid–liquid extraction was applied employing methyl-*tert*-butylether for extracting afatinib [60], erlotinib [49,51,52,56,57], everolimus [82] and gefitinib [39,40,41,42,43]. Acetonitrile-n-butylchloride 1:4 was used for extracting erlotinib [50]. Erlotinib [45] and venetoclax [96] were extracted by applying hexane-ethylacetate 1:1. In a single case, the method of LLE was not detailed [58]. Most applications had been developed for pretreating CSF samples. Everolimus was recovered from saliva [82], while, in one case, erlotinib was extracted from pleural effusate.

Solid phase extraction with a polymeric reversed-phase sorbent was employed for extracting crizotinib from CSF [71] and imatinib from leukocytes [13]. Afatinib was recovered from CSF using an octadecyl silica loading [61]. The extraction of vismodegib from CSF was feased by employing a strong mixed-mode cation exchange sorbent [94]. Gefitinib was recovered from CSF using an unspecified cartridge [44]. Equilibrium dialysis was employed to assess the unbound concentrations of ceritinib, ribociclib, and vismodegib directly in plasma [74,78,79,84].

In addition, special pretreatment procedures were described by a few authors. Imatinib was recovered from peripheral blood mononuclear cells by sonicating the defrosted pellet in an ice-water bath, followed by centrifugation, counting cells in the supernatant, washing with acetonitrile-methanol 1:1 and solvent exchange [14]. Ribociclib was assayed in CSF after dilution with methanol-water 1:1, acidification with water containing 0.2% formic acid, and centrifugation [80]. Automated sample preparation was employed as part of the analysis of everolimus [81], imatinib [18], and nilotinib [29].

Finally, afatinib and ribociclib were assayed in CSF without any sample pretreatment [59,80].

Octadecyl silica stationary phases were selected by most authors for the liquid chromatographic separation of OACDs. Octyl silica was used for the separation of imatinib, ribociclib and vemurafenib [13,16,68,80]. There are isolated examples of the application of amide (ribociclib), polystyrol-divinylbenzene (imatinib), phenyl (gefitinib), and pentafluorophenyl (ponatinib, regorafenib) phases [18,37,40,45,69,78,79,89]. In a single case of ibrutinib measurement, a nano-high performance liquid chromatography system was employed [85].

Mass spectrometric detection was performed primarily with electrospray ionization. Nevertheless, multiple examples of applying atmospheric pressure chemical ionization for the quantitation of gefitinib [39,40,42,43], imatinib [16], and erlotinib [47] in CSF were found. Bioanalytical methods developed by Bakhtiar et al., Jones et al., and Zhao et al. were adapted in these works [114,115,116]. The employed mass analyzers were triple quadrupole systems and quadrupole- linear ion trap hybrids. Ibrutinib was assayed using high-resolution mass spectrometry [85]. None of the described methods mentioned the application of negative polarity mass spectrometry. Ultraviolet–visible light absorbance detection was used for the quantitation of afatinib in CSF (254 nm) [61], erlotinib in CSF (345 nm or 348 nm) [50,51,52,56,57] and in pleural effusate (345 nm) [49], gefitinib in CSF (344 nm) [44], imatinib in CSF (260 nm) [18] and in leukocytes (261 nm) [13], as well as nilotinib (258 nm) [29] and vemurafenib (249 nm) [68] in CSF.

Most articles reported the use of an isotopically labeled internal standard when employing mass spectrometry for detection. Non-labeled substances were chosen for assaying afatinib (internal standard: imatinib) [60], dasatinib (carbamazepine and quinoxaline) [12,27], erlotinib (midazolam and desmethyl erlotinib) [45,46,47], gefitinib (vandetanib) [41], ibrutinib (propranolol) [86], imatinib (carbamazepine and quinoxaline) [12,14], nintedanib, panobinostat, regorafenib and vorinostat (carbamazepine) [12], and zanubrutinib (tolbutamide) [86]. These analyses were conducted on CSF samples, except for a single example of assaying imatinib in peripheral blood mononunclar cells [14]. The quantitation was performed without introducing an internal standard for monitoring afatinib in CSF [61], erlotinib in pleural effusate [49] and in CSF [51,52,56,57], and imatinib in breast milk [21] and in CSF [18]. UV–VIS detection was employed in all of these reports, except for one where tandem mass spectrometry was used with positive electrospray ionization [21].

Detailed information on the methods employed for monitoring OACDs in peripheral fluid spaces is provided in Table 4. Eight of the eighty-five publications (9.4%) failed to provide any methodological information or a reference to another manuscript describing the methodology employed for the quantitation of OACDs in peripheral fluid spaces. Altogether, 29 methodological publications were cited in the included manuscripts. The work of Jones et al. was cited by most included works [114]. Only five methodological works described the analysis of OACDs in peripheral fluid spaces, namely CSF (three publications), colostrum (one publication), or PBMC (one publication). The rest of the cited methodological papers described the analysis of one or more OACDs in blood.

## 4. Discussion

The rapid growth of the number of related publications reflects the increasing clinical interest in monitoring OACDs in therapeutically relevant extravascular fluids. Nevertheless, the range of substances that have been monitored in these compartments with the aim of supporting clinical decision making comprises the minor segment of marketed OACDs. Currently, imatinib is the most extensively studied drug, followed by erlotinib, gefitinib, and nilotinib. Interest in studying recently approved entities, such as dasatinib, osimertinib, panobinostat, and ribociclib, is also rising.

To date, frequently monitored peripheral fluid spaces have included cerebrospinal fluid, and, to a lesser extent, breast milk. Sporadic examples of monitoring OACDs in pleural effusion fluid, ascitic fluid, the intracellular space of peripheral blood mononuclear cells, semen, and saliva have been encountered. Collecting, handling, and processing samples originating from these fluid spaces requires expertise and, regarding CSF, pleural effusate, and ascitic fluid, specialized clinical infrastructure. Due to this limitation, as well as to the need to use specialized and resource-intensive analytical technology, it is likely that OACD monitoring in peripheral fluid spaces remains a competence of centers of excellence in oncology.

The attainment of very low OACD concentrations in CSF seems to have been unexpected by several authors. One explanation could be the poor permeability of the blood–brain barrier to these drugs, but this assumption has been contradicted by results showing that WBRT, an adjuvant intervention undertaken to increase this permeability, had not always led to increased penetration rates [67,72,91]. The application of WBRT is part of an effort to employ multimodal therapy against CNS malignancies, yet recent reports have shown that it may have detrimental adverse effects, and should not be considered as a standard measure in the therapy of NSCLC patients developing brain metastases. Experience with WBRT is also controversial regarding the treatment of primary CNS lymphomas [141]. At the same time, it has been found effective in the therapy of brain metastases of breast cancer patients, especially when combined with carboplatin injected intravenously [142]. Conventional photon radiotherapy, a similar approach with a more favorable adverse effect profile, has also been proposed for increasing the penetration rate of OACDs through the blood–brain barrier [143]. Various options of using more focal radiotherapy have also been described [144].

Another interpretation is that the CSF concentrations of OACD substances could be associated with the unbound plasma fractions. Several examples of a correlation observed between unbound serum/plasma concentrations and CSF levels confirm this assumption (Figure 3). Since the unbound fractions of various OACDs display considerable differences, it is indeed rational to judge CNS penetration based on these fractions instead of the total serum/plasma levels. The evaluation of the unbound fractions shows that the concentrations of some drugs attained in the CSF are equal to or even higher than unbound circulating concentrations. The negligible presence of everolimus in saliva, another medium accessed only by the unbound plasma fractions, confirms this rationale. No approved clinical approaches exist for establishing individual protein binding rates. Equilibrium dialysis has been used as an experimental sample pretreatment procedure for determining unbound plasma concentrations of OACDs [74,78,79,94,145]. Microdialysis has the potential to be employed for this purpose, but no examples of its application for the assessment of unbound OACD concentrations were identified. A promising sample pretreatment technology has recently become available for the rapid assessment of the extent of protein binding. The device fits into the sample preparation workflow employed by LC–MS/MS-based TDM laboratories, but there is still very limited experience regarding its use [146].

The extent of plasma protein binding may not be the only factor of the penetration of OACDs through the blood–brain barrier. Guntner et al. have shown with seven OACD substances that molecule size and the affinity of the molecule to p-glycoprotein (ABCB1 or MDR1, EC 7.6.2.2) are also key determinants. In accordance, the permeability of the blood–brain barrier to dasatinib, imatinib, regorafenib, ribociclib, and vorinostat was higher than to nintedanib or panobinostat. The comparison of experimental results to those obtained using computer models nevertheless indicated that further variables, currently unidentified, are likely to play an important role in this process [12].

In sum, more research is needed to find dosages and monitoring approaches that result in the attainment of clinically sufficient CSF concentrations in all patients. Aggressive dosing, the artificial facilitation of the penetration of drugs through the blood–brain barrier, or the administration of drug combinations containing a component which inhibits p-glycoprotein or other drug-eliminating proteins relevant to a specific OACD are potential strategies for the more efficient therapy of CNS malignancies. A methodology is also emerging to predict OACD treatment efficacy by comparing the drug concentrations measured in the target peripheral fluid to the in vitro IC_50_ established for the given malignant cell line, and based on this relationship, by creating a mathematical link between the pharmacokinetic and pharmacodynamic properties of the administered drug. In the future, this approach may prove useful in developing precision dosing schemes with pharmacokinetic–pharmacodynamic targets, in an analogy to those already employed for guiding antibiotic therapy. 

In sharp contrast to the observations made in the CSF, high penetration rates or even the accumulation of OACDs were consistently described in exudates formed by pleural effusion and malignant ascites, and in excreta such as breast milk and semen. Imatinib showed considerable accumulation in buffy coat cells and in peripheral blood mononuclear cells. These findings indicate that the consideration of third spaces as pharmacokinetic compartments may be rational in patients treated with lung or breast cancer, as well as in leukemia patients.

There has been a solid consensus in relying on liquid chromatography-based analytical approaches for the therapeutic monitoring of OACDs in peripheral fluid spaces. Several early methods relied on the use of UV–VIS detectors, but LC–MS/MS has by now emerged as the primary analytical technique as a result of ensuring sufficient selectivity and sensitivity, requiring small sample volumes for the analysis, and allowing the high-throughput processing of peripheral fluid space samples. When applied for the clinical analysis of OACDs in these compartments, the main steps of these methodologies were reversed phase chromatographic separation followed by positive electrospray ionization and multiple reaction monitoring. The simple and rapid process of deproteinization was in most cases sufficient for the pretreatment of samples. A common weakness of the analytical methodologies employed in the reviewed records is that they had not undergone comprehensive validation, lowering the credibility of the presented results.

The application of equilibrium dialysis to retrieve direct clinical pharmacological information fits into a series of related emerging approaches, such as the rapid assessment of protein binding, or the partitioning between plasma and red blood cells. Such technologies are expected to facilitate the reporting of truly individualized, and, in a clinical sense, substantially more relevant information on the pharmacokinetic properties of drugs including OACDs in the future [146,147].

An important limitation of the performed evaluation is that only a minority of the retrieved publications described the outcomes of registered clinical trials. The majority of the works reported small-scale, researcher-initiated, unicentric studies, case series, or case reports. In addition, the methodologies employed for sample collection and analysis were uniquely developed by most investigators, limiting the comparability of results. Only a fraction of the subjects involved in the studies had given their consent for collecting CSF samples; consequently, the number of available CSF concentrations was small in several publications. Indeed, peripheral space drug monitoring was conducted as a collateral tool of diagnosis or patient status monitoring in several cases.

Malignancies are the leading causes of premature death worldwide, with breast and lung cancers underlying the largest number of new cases [148]. The importance of improving the treatment of these diseases is therefore beyond dispute. Therapeutic drug monitoring and research regarding model-informed precision dosing is currently based on the evaluation of drug concentrations in the systemic circulation, while evidence now shows that the monitoring of OACDs in therapeutically relevant extravascular fluid compartments can be equally important, especially for the better treatment of central nervous system malignancies. TDM laboratories providing service for large oncological centers can add a fundamental impetus by introducing suitable, validated, LC–MS/MS-based analytical methods for monitoring these drugs in peripheral fluid spaces, and in vitro approaches to determining unbound OACD concentrations. Establishing these competences is the first step for the introduction of therapy guidance based on highly relevant pharmacokinetic models and pharmacokinetic–pharmacodynamic indices, as well as for the early detection of suboptimal dosages and the risk of certain adverse effects. Since the number of available OACDs, as well as the range of their indications, is growing rapidly, the identification of further therapeutic goals and therapeutically relevant peripheral fluid spaces can be expected, maintaining a long-term need for the close cooperation of clinicians, clinical pharmacologists, and the TDM service in this field.

## 5. Conclusions

This review has revealed that the therapeutic monitoring of OACDs in peripheral fluid spaces is an important diagnostic tool for the assessment of the penetration of these substances into CSF and third space fluids, which is imperative for the optimization of drug administration, and of their appearance in excreta, which may convey important information on adverse effects and other forms of toxicity. LC–MS/MS is an established analytical technology for performing these measurements, with little effort required to transfer conventional, blood-based TDM methods. Nevertheless, dedicated centers of excellence are needed to perform such measurements routinely.

A range of indications has been identified for which the TDM of OACDs in peripheral fluid spaces can provide clinically powerful information. More systematic studies with rigorous quality control are needed, however, for elucidating the pharmacokinetic properties of OACDs, for setting quantitative therapeutic targets, and for establishing standard analytical methodology. Related research in pediatric populations still remains an unmet need.

After more than 20 years of using OACDs, an alarmingly small number of these substances has ever been investigated in a clinically important peripheral fluid space. In several malignancies, the administration of these medications cannot be optimized without knowledge regarding their quantities in these fluid spaces, especially in CSF; therefore, research should be focused on gathering information on all OACDs in this respect.

## Figures and Tables

**Table 1:** Orally taken, small-molecule anticancer medications with specific cellular protein targets which have been measured in peripheral fluid spaces to support clinical decision making. CSF, cerebrospinal fluid; PBMC, peripheral blood mononuclear cells.

ATC Code	International Nonproprietary Name	Target Cellular Protein	Monitored Peripheral Fluid	Ref.
L01EA01	Imatinib	BCR-ABL tyrosine kinase	CSF, breast milk, leukocytes, PBMC, semen	[12,13,14,15,16,17,18,19,20,21,22,23,24,25,26]
L01EA02	Dasatinib	BCR-ABL tyrosine kinase	CSF	[12,27,28]
L01EA03	Nilotinib	BCR-ABL tyrosine kinase	CSF, pleural fluid	[29,30,31,32,33,34,35,36]
L01EA05	Ponatinib	BCR-ABL tyrosine kinase	CSF	[37]
L01EB01	Gefitinib	Epidermal growth factor receptor tyrosine kinase	CSF, pleural fluid, peritoneal dialysis fluid	[38,39,40,41,42,43,44,45,46]
L01EB02	Erlotinib	Epidermal growth factor receptor tyrosine kinase	CSF, pleural fluid	[45,46,47,48,49,50,51,52,53,54,55,56,57,58]
L01EB03	Afatinib	Epidermal growth factor receptor tyrosine kinase	CSF	[59,60,61]
L01EB04	Osimertinib	Epidermal growth factor receptor tyrosine kinase	CSF	[62,63,64,65]
L01EB08	Icotinib	Epidermal growth factor receptor tyrosine kinase	CSF	[66,67]
L01EC01	Vemurafenib	B-raf serine-threonine kinase	CSF	[68]
L01EC02	Dabrafenib	B-raf serine-threonine kinase	CSF	[69]
L01ED01	Crizotinib	Anaplastic lymphoma kinase	CSF	[70,71,72,73]
L01ED02	Ceritinib	Anaplastic lymphoma kinase	CSF	[74]
L01ED03	Alectinib	Anaplastic lymphoma kinase	CSF	[75,76]
L01ED05	Lorlatinib	Anaplastic lymphoma kinase	CSF	[77]
L01EE01	Trametinib	Mitogen-activated protein kinase	CSF	[69]
L01EF02	Ribociclib	Cycline dependent kinase	CSF	[12,78,79,80]
L01EG02	Everolimus	Mammalian target of rapamycin	breast milk, saliva	[81,82]
L01EH01	Lapatinib	Human epidermal growth factor receptor 2 tyrosine kinase	CSF	[83]
L01EH02	Neratinib	Human epidermal growth factor receptor 2 tyrosine kinase	CSF	[84]
L01EL01	Ibrutinib	Bruton’s tyrosine kinase	CSF	[85,86]
L01EL03	Zanubrutinib	Bruton’s tyrosine kinase	CSF	[87]
L01EX01	Sunitinib	Other protein kinase	Ascitic fluid	[88]
L01EX03	Pazopanib	Other protein kinase	Ascitic fluid	[88]
L01EX05	Regorafenib	Other protein kinase	CSF	[12,89]
L01EX09	Nintedanib	Other protein kinase	CSF	[12]
L01EX21	Tepotinib	Other protein kinase	CSF	[90,91]
L01EX23	Pralsetinib	Other protein kinase	CSF	[65]
L01XH01	Vorinostat	Histone deacetylase	CSF	[12]
L01XH03	Panobinostat	Histone deacetylase	CSF	[12,92,93]
L01XJ01	Vismodegib	Hedgehog pathway proteins	CSF	[94]
L01XX52	Venetoclax	bcl-2 protein	CSF	[95,96]

**Table 2:** Characteristics of the included articles. ALL, acute lymphocytic leukemia. AML, acute myeloid leukemia. CLL, chronic lymphocytic leukemia; CML, chronic myeloid leukemia; CML-BC, chronic myeloid leukemia with blast crisis; CNS, central nervous system; CSF, cerebrospinal fluid; HIV, human immunodeficiency virus; LM, leptomeningeal metastasis; NSCLC, non-small cell lung cancer; PBMC, peripheral blood mononuclear cell; PFS, peripheral fluid sample; Ph +, Philadelphia chromosome-positive.

First Author, Year	Drug	Type of Study	Patient Population	Number of Subjects Donating Samples for TDM	Period of Recruitment	Outcomes Measured	Ref.
Hoffknecht, 2015	Afatinib	Case series	2 adults with advanced NSCLC with brain metastasis or leptomeningeal disease	Blood: 2 PFS: 2	May 2010 to December 2013	Afatinib in CSF and in plasma	[59]
Kawaguchi, 2017	Afatinib	Case report	Adult (female, 41 years) with stage IV lung adenocarcinoma and cerebral metastasis	Blood: 1 PFS: 1	Not applicable	Afatinib in CSF and in plasma	[60]
Tamiya, 2017	Afatinib	Registered clinical study (UMIN000014065)	11 adults with histologically proven EGFR mutation-positive NSCLC with LMC	Blood: 11 PFS: 8	April 2014 to November 2015	Afatinib in CSF and in plasma	[61]
Gadgeel, 2014	Alectinib	Registered clinical study (NCT01588028)	47 adults with locally advanced or metastatic NSCLC with ALK gene rearrangement	Blood: 5 PFS: 5	3 May 2012 to 26 July 2013	Alectinib in CSF and in plasma	[75]
Metro, 2016	Alectinib	Case series	11 ALK-positive NSCLC patients with CNS metastasis	Blood: 2 PFS sample: 2	December 2013 to August 2015	Alectinib in CSF and in serum	[76]
Mehta, 2021	Ceritinib	Registered clinical study (NCT02605746)	10 adults with glioblastoma necessitating resection	Blood: 10 PFS: 8	Not reported	Ceritinib in CSF and in plasma	[74]
Costa, 2011	Crizotinib	Case report	Adult (male, 29 years) with stage IV NSCLC and CNS metastasis	Blood: 1 PFS: 1	Not applicable	Crizotinib in CSF and in plasma	[70]
Metro, 2015	Crizotinib	Case series	2 adults with ALK-positive advanced NSCLC and CNS metastasis	Blood: 2 PFS: 2	Not applicable	Crizotinib in CSF and in plasma	[71]
Okawa, 2018	Crizotinib	Case report	Adult (male, 60 years) with NSCLC and isolated CNS failure	Blood: 1 PFS: 1	Not applicable	Crizotinib in CSF and in plasma	[72]
Okimoto, 2019	Crizotinib	Case report	Adult (male, 61 years) with NSCLC and carcinomatous meningitis	Blood: 1 PFS: 1	Not applicable	Crizotinib in CSF and in plasma	[73]
Hottinger, 2019	Dabrafenib, trametinib	Case series	2 adults with leptomeningeal tumor	Blood: 2 PFS: 2	2017	Dabrafenib and trametinib in CSF and in plasma	[69]
Guntner, 2020	Dasatinib, imatinib, nintedanib, panobinostat, regorafenib, ribociclib, vorinostat	Case series	12 pediatric patients (ages: 7.5–20.3) with primary and secondary malignant brain tumors	Blood: 1 PFS: 9	Not reported	Imatinib, dasatinib, nintedanib, panobinostat, regorafenib, ribociclib and vorinostat in CSF	[12]
Kondo, 2014	Dasatinib	Case report	Adult (female, 58 years) with Ph + ALL and meningeal leukemia	Blood: 1 PFS: 1	Not applicable	Dasatinib in CSF and in plasma	[27]
Gong, 2021	Dasatinib	Registered clinical study (NCT02523976)	31 adults with newly diagnosed Ph + ALL	Blood: 31 PFS: 31	January 2016 to April 2018	Dasatinib in CSF and in plasma	[28]
Shriyan, 2020	Erlotinib, gefitinib	Non-registered, researcher-initiated study	20 adults with NSCLC and brain metastasis	Blood: 20 PFS: 20	August 2014 to July 2017	Erlotinib in CSF and in plasma, gefitinib in CSF and in plasma	[46]
Broniscer, 2007	Erlotinib	Case report	Pediatric patient (female, 8 years) with glioblastoma	Blood: 1 PFS: 1	Not applicable	Erlotinib in CSF and in plasma	[47]
Rogers, 2010	Erlotinib	Case report	Adult (female, 33 years) with CNS hemangioblastomatosis associated with von Hippel-Lindau disease	Blood: 1 PFS: 1	Not reported	Erlotinib in CSF and in plasma	[48]
Masago, 2011	Erlotinib	Non-registered, researcher-initiated study	9 adult patients with advanced NSCLC	Blood: 9 PFS: 9	June 2009 to December 2009	Erlotinib and OSI-420 in pleural effusate and in plasma	[49]
Masuda, 2011	Erlotinib	Case series	3 adults (NSCLC with LM)	Blood: 3 PFS: 3	Not applicable	Erlotinib in CSF and in plasma	[50]
Togashi, 2010	Erlotinib	Case series	4 adults with NSCLC and CNS metastasis	Blood: 4 PFS: 4	Not reported	Erlotinib in CSF and in plasma	[51]
Deng, 2013	Erlotinib	Non-registered, researcher-initiated study	6 adults (NSCLC)	Blood: 6 PFS: 6	March 2011 to March 2012	Erlotinib in CSF and in plasma	[52]
Sakata, 2016	Erlotinib	Case report	Adult (female, 54 years) with NSCLC and LM	Blood: 1 PFS: 1	Not applicable	Erlotinib in CSF and in plasma	[53]
Clarke, 2010	Erlotinib	Case report	Adult (female, 54 years) with stage IV NSCLC and LM	Blood: 1 PFS: 1	Not applicable	Erlotinib in CSF and in plasma	[54]
Yang, 2015	Erlotinib	Retrospective observational study	9 adults with lung adenocarcinoma and refractory CNS metastases	Blood: 6 PFS: 6	January 2011 to June 2013	Erlotinib in CSF and in plasma	[55]
Togashi, 2011	Erlotinib	Case series	9 adults with NSCLC and CNS metastasis	Blood: 9 PFS: 9	Not reported	Erlotinib in CSF and in plasma	[56]
Fukudo, 2013	Erlotinib	Non-registered, researcher-initiated study	88 adults with NSCLC	Blood: 88 PFS: 23	June 2009 to March 2012	Erlotinib in CSF and in plasma	[57]
Nosaki, 2020	Erlotinib	Registered clinical study (UMIN000007020)	21 adults (stage IV NSCLC or its recurrence with LM)	Blood: 14 PFS: 12	December 2011 to May 2015	Erlotinib in CSF and in plasma	[58]
DeWire, 2021	Everolimus, ribociclib	Registered clinical study (NCT03387020)	22 pediatric patients (ages: 3.9–20.4) with a recurrent, progressive or refractory brain tumor	Blood: 22 PFS: 5	January 2018 to April 2020	Ribociclib in CSF and in plasma, everolimus in blood	[80]
Fiocchi, 2016	Everolimus	Case report	Adult (female, 40 years) with pregnancy after undergoing heart transplant	Blood: 1 PFS: 1	Not applicable	Everolimus in breast milk (colostrum) and in plasma	[81]
Molenaar-Kuijsten, 2022	Everolimus	Registered clinical study (EudraCT 2014-004,833-25; NTR4908)	10 adults with stomatitis	Blood: 10 PFS: 10	Not reported	Everolimus in saliva and in plasma	[82]
Yamaguchi, 2015	Gefitinib	Case report	Adult (male, 72 years) lung adenocarcinoma and brain metastasis	Blood: 1 PFS: 1	Not applicable	Gefitinib in pleural effusate, peritoneal effusate dialysate, and plasma	[38]
Fukuhara, 2008	Gefitinib	Case report	Adult (male, 62 years) with stage IV lung cancer and carcinomatous meningitis	Blood: 1 PFS: 1	Not applicable	Gefitinib in CSF and in plasma	[39]
Zhao, 2013	Gefitinib	Non-registered, researcher-initiated study	22 adults (NSCLC)	Blood: 22 PFS: 22	March 2007 to December 2010	Gefitinib in CSF and in plasma	[40]
Zeng, 2015	Gefitinib	Non-registered, researcher-initiated study	28 adults with NSCLC and brain metastasis	Blood: 28 PFS: 28	October 2009 to March 2011	Gefitinib in CSF and in plasma	[41]
Zhao, 2016	Gefitinib	Case series	7 adults with NSCLC with intracranial and/or extracranial progression	Blood: 5 PFS: 5	February 2009 to May 2013	Gefitinib in CSF and in plasma	[42]
Jackman, 2015	Gefitinib	Registered clinical study (NCT00372515)	7 adults with NSCLC and LM	Blood: 7 PFS: 7	May 2006 and July 2008	Gefitinib in CSF and in plasma	[43]
Fang, 2015	Gefitinib	Case series	3 adults with lung adenocarcinoma and brain metastasis	Blood: 3 PFS: 3	Not reported	Gefitinib in CSF and in plasma	[44]
Togashi, 2012	Gefitinib, erlotinib	Non-registered, researcher-initiated study	15 adults (NSCLC with CNS metastases with EGFR mutations)	Gefitinib: Blood: 8 PFS: 8 Erlotinib: Blood: 9 PFS: 9	April 2010 to March 2012	(1) Gefitinib in CSF and in plasma; (1) Erlotinib in CSF and in plasma	[45]
Law, 2021	Ibrutinib	Case series	2 adults with Epstein–Barr virus-associated primary CNS lymphoma	Blood: 1 PFS: 1	Not applicable	Ibritunib in CSF and in plasma	[85]
Yu, 2021	Ibrutinib	Retrospective observational study	3 adults with primary central nervouos system lymphoma	Blood: 3 PFS: 1	August 2017 to May 2020	Ibrutinib in CSF and in plasma	[86]
Fan, 2015	Icotinib	Registered clinical study (NCT01514877)	20 adults with NSCLC and brain metastasis	Blood: 10 PFS: 10	February 2012 to March 2013	Icotinib in CSF and in plasma	[66]
Zhou, 2016	Icotinib	Registered clinical study (NCT01516983)	15 adults with NSCLC and brain metastasis	Blood: 15 PFS: 13	13 February 2012 to 24 July 2013	Icotinib in CSF and in plasma	[67]
Nambu, 2011	Imatinib	Non-registered, researcher-initiated study	15 adults with CML	Blood: 15 PFS: 15	2003 to 2008	Imatinib in leukocytes and in plasma	[13]
De Francia, 2014	Imatinib	Non-registered, researcher-initiated study	24 adults with Ph + CML	Blood: 24 PFS: 24	Not reported	Imatinib in PBMC’s and in plasma	[14]
Petzer, 2002	Imatinib	Case report	Adult (male, 52 years) with CML with CNS relapse	Blood: 1 PFS: 1	Not applicable	Imatinib in CSF and in plasma	[15]
Takayama, 2002	Imatinib	Case report	Adult (female, 32 years) with Ph + ALL and CNS leukemia	Blood: 1 PFS: 1	Not applicable	Imatinib in CSF and in plasma	[16]
Bornhauser, 2004	Imatinib	Case report	Adult (female, 56 years) with Ph + CML and CNS leukemia	Blood: 1 PFS: 1	Not applicable	Imatinib and N-desmethyl imatinib in CSF and in plasma	[17]
le Coutre, 2004	Imatinib	Non-registered, researcher-initiated study	97 subjects with BCR/ABL + CML or BCR/ABL + ALL	Blood: 97 PFS: 17	Not reported	Imatinib and N-desmethyl imatinib in CSF and in plasma	[18]
Leis, 2004	Imatinib	Registered clinical study (CSTI5710102, CSTI15710109	42 adults with CML in blast crisis, or Ph + ALL	Blood: 4 PFS: 4	Not reported	Imatinib in CSF and in plasma	[19]
Russell, 2007	Imatinib	Case series	2 adults with Ph + CML	Blood: 1 PFS: 1	Not applicable	Imatinib in breast milk and in plasma	[20]
Gambacorti-Passerini, 2007	Imatinib	Case report	Adult (female, 40 years) with CML	Blood: 1 PFS: 1	Not applicable	Imatinib in breast milk and in plasma	[21]
Ali, 2009	Imatinib	Case report	Adult (female, 27 years) with Ph + CML	Blood: 1 PFS: 1	Not applicable	Imatinib in breast milk and in plasma	[22]
Kronenberger, 2009	Imatinib	Case report	Adult (female, 34 years) with CML	Blood: 1 PFS: 1	Not applicable	Imatinib in breast milk and in plasma	[23]
Burwick, 2017	Imatinib	Case report	Adult (female, 29 years) with Ph + CML	Blood: 1 PFS: 1	Not applicable	Imatinib in breast milk and in plasma	[24]
Terao, 2020	Imatinib	Case report	Adult (female, 32 years) with Ph + CML	Blood: 1 PFS: 1	Not applicable	Imatinib in breast milk and in plasma	[25]
Chang, 2017	Imatinib	Non-registered, researcher-initiated study	108 males (15–51 years) with CML-CP, infertility, or controls	Blood: 48 PFS: 11	January 2010 to December 2014	Imatinib in semen and in plasma	[26]
Gori, 2014	Lapatinib	Case series	2 adults with HER2 + metastatic breast cancer	Blood: 2 PFS: 2	Not applicable	Lapatinib in CSF and in plasma	[83]
Sun, 2022	Lorlatinib	Registered clinical study (NCT01970865)	54 patients with NSCLC and suspected or confirmed leptomeningeal carcinomatosis or carcinomatous meningitis	Blood: 54 PFS: 5	Not reported	Lorlatinib in CSF and in plasma	[77]
Freedman, 2020	Neratinib	Registered clinical study (NCT01494662)	5 adults with HER2 + breast cancer and brain metastases in whom craniotomy was indicated	Blood: 2 PFS: 3	22 May 2013 to 18 October 2016	Neratinib in CSF and in plasma	[84]
Reinwald, 2014	Nilotinib	Case series	4 patients aged > 15 years with BCR-ABL + ALL or CML-BC	Blood: 4 PFS: 4	Not reported	Nilotinib in CSF and in plasma	[29]
Liu, 2019	Nilotinib	Registered clinical study (ChiCTR-ONC-12002469)	30 subjects aged > 15 years with newly diagnosed Ph + ALL	Blood: 30 PFS: 30	14 September 2011 to 21 November 2013	Nilotinib in CSF and in plasma	[30]
Satoh, 2021	Nilotinib	Case report	Adult (male, 23 years) with CML	Blood: 1 PFS: 1	Not applicable	Nilotinib in pleural effusate and in plasma	[31]
Pagan, 2016	Nilotinib	Registered clinical study (NCT02281474)	12 adults with Parkinson’s disease or Dementia with Lewy Bodies	Blood: 12 PFS: 12	Not reported	Nilotinib in CSF and in plasma	[32]
Pagan, 2019	Nilotinib	Registered clinical study (NCT02954978)	75 adults with Parkinson’s disease	Blood: 75 PFS: 75	Not reported	Nilotinib in CSF and in plasma	[33]
Pagan, 2020	Nilotinib	Registered clinical study(NCT02954978)	75 adults with Parkinson’s disease	Blood: 75 PFS: 75	17 May 2017 to 28 April 2018	Nilotinib in CSF and in plasma	[34]
Simuni, 2021	Nilotinib	Registered clinical study (NCT03205488)	76 adults with Parkinson’s disease	Blood: 41 PFS: 42	November 2017 to December 2018	Nilotinib in CSF and in plasma	[35]
Turner, 2020	Nilotinib	Registered clinical study (NCT02947893)	37 adults with Alzheimer’s disease	Blood: 37 PFS: 37	Not reported	Nilotinib in CSF and in plasma	[36]
Song, 2019	Osimertinib	Case report	Adult with NSCLC and LM	Blood: 1 PFS: 1	Not applicable	Osimertinib in CSF and in plasma	[62]
Xing, 2020	Osimertinib	Registered clinical study (NCT02972333)	38 adults with refractory NSCLC and CNS metastasis	Blood: 12 PFS: 12	January 2017 to September 2017	Osimertinib in CSF and in plasma	[63]
Yamaguchi, 2021	Osimertinib	Registered clinical study (UMIN000024218, jRCTs071180017)	40 adults with confirmed NSCLC and CNS metastasis	Blood: 37 PFS: 7	27 December 2016 to 4 July 2019	Osimertinib in CSF and in plasma	[64]
Rasmussen, 2015	Panobinobstat	Registered clinical study (NCT01680094)	15 adults with HIV infection	Blood: 0 PFS: 11	September 2012 to February 2014	Panobinostat in CSF	[92]
Goldberg, 2020	Panobinostat	Registered clinical study (NCT01321346)	22 pediatric patients with relapsed or refractory acute leukemia or lymphoma	Blood: 9 PFS: not reported	3 November 2011 to 31 July 2015	Panobinostat in CSF and in plasma	[93]
Krens, 2021	Pazopanib	Case report	Adult (male, 79 years) with metastatic papillary renal cell carcinaoma and malignant ascites	Blood: 1 PFS: 1	Not applicable	Pazopanib in ascitic fluid and in plasma	[88]
Tanimura, 2021	Ponatinib	Case report	Pediatric patient (girl, 3 years) with Ph + ALL and CNS infiltration	Blood: 1 PFS: 1	Not applicable	Ponatinib in CSF and in plasma	[37]
Zhao, 2022	Pralsetinib	Case report	Adult (female, 43 years) with lung cancer and meningeal metastases	Blood: 1 PFS: 1	Not applicable	Pralsetinib in CSF and in plasma	[65]
Zeiner, 2019	Regorafenib	Retrospective observational study	21 adults with recurrent malignant glioma	Blood: 3 PFS: 3	August 2018 to July 2019	Regorafenib in CSF and in serum	[89]
Miller, 2019	Ribociclib	Registered clinical tudy (NCT02345824, IND125168)	3 adults with recurrent glioblastoma	Blood: 3 PFS: 1	First surgery dates: 29 March 2012 to 26 September 2014	Ribociclib in CSF and in plasma	[78]
Tien, 2019	Ribociclib	Registered clinical study (NCT02933736)	12 adults with a recurrent glioblastoma	Blood: 12 PFS: 12	Not reported	Ribociclib in CSF and in plasma	[79]
Tanaka, 2020	Tepotinib	Case report	Adult (male, 56 years) with with lung adenocarcinoma and LM	Blood: 1 PFS: 1	Not applicable	Tepotinib in CSF and in plasma	[90]
Ninomaru, 2021	Tepotinib	Case report	Adult (female, 77 years) with NSCLC and LM	Blood: 1 PFS: 1	Not applicable	Tepotinib in CSF and in plasma	[91]
Sakji-Dupré, 2015	Vemurafenib	Case series	6 adults with melanoma and brain metastasis	Blood: 6 PFS: 6	February 2012 to January 2013	Vemurafenib in CSF and in plasma	[68]
Reda, 2019	Venetoclax	Case report	Adult (male, 58 years) with trisomy 12, IGHV unmutated (VH4L) chronic lymphocytic leukemia	Blood: 1 PFS: 1	Not applicable	Venetoclax in CSF and in plasma	[95]
Condorelli, 2022	Venetoclax	Case report	Adult (male, 52 years) with AML and CNS leukemia	Blood: 1 PFS: 1	Not applicable	Venetoclax in CSF and in plasma	[96]
Gajjar, 2013	Vismodegib	Registered clinical study (NCT0082248)	33 pediatric patients (ages: 3.9–21 yeatrs) with recurrent, progressive or refractory medulloblastoma	Blood: 33 PFS: 3	Not reported	Vismodegib in CSF and in plasma	[94]
Zhang, 2021	Zanubrutinib	Case series	13 adults with diffuse large B-cell lymphoma and CNS involvement	Blood: 13 PFS: 13	August 2020 to December 2020	Zanubrutinib in CSF and in plasma	[87]

**Table 3:** Clinical background of the monitoring of OACDs in peripheral fluid spaces. ALL, acute lymphoid leukemia; AML, acute myeloid leukemia; CNS, central nervous system; CSF, cerebrospinal fluid; CLL, chronic lymphoid leukemia; CML, chronic myeloid leukemia; NSCLC, non-small cell lung cancer; PBMC, peripheral blood mononuclear cells; PCNSL, primary central nervous system lymphoma.

International Non-Proprietary Name	Peripheral Compartment	Indication of Monitoring	Pathological Condition	Ref.
Afatinib	CSF	Secondary malignancy	NSCLC with CNS metastasis and/or leptomeningeal disease	[59]
	CSF	Secondary malignancy	NSCLC with leptomeningeal carcinomatosis	[60]
	CSF	Secondary malignancy	NSCLC with leptomeningeal carcinomatosis	[61]
Alectinib	CSF	Secondary malignancy	NSCLC with CNS metastasis and systemic disease	[75]
	CSF	Secondary malignancy	NSCLC with CNS metastasis	[76]
Ceritinib	CSF	Secondary malignancy	CNS metastasis of breast tumor, head and neck tumor or melanoma, recurrent glioblastoma	[74]
Crizotinib	CSF	Secondary malignancy	NSCLC with leptomeningeal metastasis	[70]
	CSF	Secondary malignancy	NSCLC with CNS metastasis	[71]
	CSF	Secondary malignancy	NSCLC with CNS metastasis	[72]
	CSF	Secondary malignancy	NSCLC with carcinomatous meningitis	[73]
Dabrafenib	CSF	Primary malignancy	Glioma	[69]
Dasatinib	CSF	Primary malignancy	CNS tumor	[12]
	CSF	Secondary malignancy	AML with extramedullary and meningeal relapse	[27]
	CSF	Secondary malignancy	ALL and CNS leukemia prophylaxis	[28]
Erlotinib	CSF	Secondary malignancy	NSCLC with CNS metastasis	[45]
	CSF	Secondary malignancy	NSCLC with CNS metastasis	[46]
	CSF	Primary malignancy	Glioblastoma	[47]
	CSF	Primary malignancy	CNS hemangioblastoma with von Hippel-Lindau disease	[48]
	CSF	Secondary malignancy	NSCLC with leptomeningeal metastasis	[50]
	CSF	Secondary malignancy	NSCLC with CNS metastasis	[51]
	CSF	Secondary malignancy	NSCLC with CNS metastasis	[52]
	CSF	Secondary malignancy	NSCLC with leptomeningeal metastasis	[53]
	CSF	Secondary malignancy	NSCLC with leptomeningeal meatastasis	[54]
	CSF	Secondary malignancy	NSCLC with refractory CNS metastasis	[55]
	CSF	Secondary malignancy	NSCLC with CNS metastasis	[56]
	CSF	Secondary malignancy	NSCLC with CNS metastasis	[57]
	CSF	Secondary malignancy	NSCLC with leptomeningeal metastasis	[58]
	Pleural effusate	Primary malignancy	NSCLC	[49]
Everolimus	Breastmilk	Risk of toxicity	Pregnancy in everolimus-treated heart-transplanted patient	[81]
	Saliva	Risk of toxicity	Cancer patients (breast, renal cell, neuroendocrine tumors)	[82]
Gefitinib	CSF	Secondary malignancy	NSCLC with carcinomatous meningitis	[39]
	CSF	Secondary malignancy	NSCLC with lung adenocarcinoma	[40]
	CSF	Secondary malignancy	NSCLC with CNS metastasis	[41]
	CSF	Secondary malignancy	NSCLC with leptomeningeal metastasis	[42]
	CSF	Secondary malignancy	NSCLC with leptomeningeal metastasis	[43]
	CSF	Secondary malignancy	NSCLC with CNS metastasis	[44]
	CSF	Secondary malignancy	NSCLC with CNS metastasis	[45]
	CSF	Secondary malignancy	NSCLC with CNS metastasis	[46]
	Pleural effusate, peritoneal effusate dialysate	Primary malignancy	NSCLC	[38]
Ibrutinib	CSF	Primary malignancy	Epstein–Barr associated primary CNS lymphoma	[85]
	CSF	Primary malignancy	PCNSL	[86]
Icotinib	CSF	Secondary malignancy	NSCLC with CNS metastasis	[44]
	CSF	Secondary malignancy	NSCLC with CNS metastatis	[67]
Imatinib	Breast milk	Risk of toxicity	CML during pregnancy	[20]
	Breast milk	Risk of toxicity	CML during pregnancy	[21]
	Breast milk	Risk of toxicity	CML during pregnancy and breastfeeding	[22]
	Breast milk	Risk of toxicitiy	CML during pregnancy and breastfeeding	[23]
	Breast milk	Risk of toxicity	CML in early pregnancy and breastfeedng	[24]
	Breast milk	Risk of toxicity	CML during pregnancy and breastfeeding	[25]
	CSF	Primary malignancy	CNS tumor	[12]
	CSF	Secondary malignancy	CML with lymphoid blast crisis	[15]
	CSF	Secondary malignancy	ALL with CNS leukemia	[16]
	CSF	Secondary malignancy	CML with CNS leukemia	[17]
	CSF	Secondary malignancy	CML and ALL with meningeous leukemia	[18]
	CSF	Secondary malignancy	CML with lymphoid blast crisis and AML	[19]
	Leukocytes	Primary malignancy	CML	[13]
	PBMC’s	Primary malignancy	CML	[14]
	Semen	Risk of toxicity	CML	[26]
Lapatinib	CSF	Secondary malignancy	Breast cancer with CNS metastasis	[83]
Lorlatinib	CSF	Secondary malignancy	NSCLC with CNS metastasis	[77]
Neratinib	CSF	Secondary malignancy	Breast cancer with CNS metastasis	[84]
Nilotinib	CSF	Secondary malignancy	Leukemia with CNS infiltration	[29]
	CSF	Secondary malignancy	ALL and CNS leukemia prophylaxis	[30]
	CSF	Treatment of a mental disorder	Parkinson’s disease, dementia	[32]
	CSF	Treatment of a mental disorder	Parkinson’s disease	[33]
	CSF	Treatment of a mental disorder	Parkinson’s disease	[34]
	CSF	Treatment of a mental disorder	Parkinson’s disease	[35]
	CSF	Treatment of a mental disorder	Alzheimer’s disease	[36]
	Pleural effusate	Risk of toxicity	CML	[31]
Nintedanib	CSF	Primary malignancy	CNS tumor	[12]
Osimertinib	CSF	Secondary malignancy	NSCLC with leptomeningeal metastasis	[62]
	CSF	Secondary malignancy	NSCLC wth CNS metastasis	[63]
	CSF	Secondary malignancy	NSCLC with CNS metastasis	[64]
	CSF	Secondary malignancy	NSCLC with meningeal metastasis	[65]
Panobinostat	CSF	Primary malignancy	CNS tumor	[12]
	CSF	Risk of toxicity	HIV infection	[92]
	CSF	Risk of toxicity	Recurrent or refractory haematologic malignancies (leukemia and lymphoma)	[93]
Pazopanib	Ascitic fluid	Secondary malignancy	Metastatic papillary renal cell carcinoma and malignant ascites	[88]
Ponatinib	CSF	Secondary malignancy	ALL with CNS leukemia	[37]
Pralsetinib	CSF	Secondary malignancy	NSCLC with meningeal metastasis	[65]
Regorafenib	CSF	Primary malignancy	CNS tumor	[12]
	CSF	Primary malignancy	Recurrent malignant glioma	[89]
Ribociclib	CSF	Primary malignancy	CNS tumor	[12]
	CSF	Primary malignancy	Recurrent glioblastma	[78]
	CSF	Primary malignancy	Recurrent glioblastoma	[79]
	CSF	Primary malignancy	Recurrent or refractory malignant CNS tumor	[80]
Sunitinib	Ascitic fluid	Secondary malignancy	Metastatic papillary renal cell carcinoma and malignant ascites	[88]
Tepotinib	CSF	Secondary malignancy	NSCLC with leptomeningeal metastasis	[90]
	CSF	Secondary malignancy	NSCLC with leptomeningeal metastasis	[91]
Trametinib	CSF	Primary malignancy	Glioma	[69]
Vemurafenib	CSF	Secondary malignancy	Melanoma with CNS metastasis	[68]
Venetoclax	CSF	Secondary malignancy	CLL with CNS involvement	[95]
	CSF	Secondary malignancy	AML with leptomeningeal involvement	[96]
Vismodegib	CSF	Primary malignancy	Recurrent or refractory medulloblastoma	[94]
Vorinostat	CSF	Primary malignancy	CNS tumor	[12]
Zanubrutinib	CSF	Primary malignancy	CNS lymphoma	[87]

**Table 4:** Analytical approaches to monitoring the concentrations of orally administered, small-molecule anticancer medications with tumor-specific cellular protein targets in peripheral fluid spaces. CSF, cerebrospinal fluid; Deprot., deproteinization; FA, formic acid; IS, internal standard; LLE, liquid–liquid extraction; NS, not specified; PBMC, peripheral blood mononuclear cells; SPE, solid phase extraction; TFA, trifluoroacetic acid.

Drug	Matrix	Internal Standard	Chromatography			Mass Spectrometry			UV–VIS (nm)	Sample Preparation	Ref.
			Stationary Phase	Mobile Phases	Type of Separation	Ioniza-tion	Analyte ions	IS Ions			
Afatinib	CSF	Isotope-labeled afatinib	Reversed phase	NS	Gradient	ESI (+)	NS	NS	Not used	None	[59]
Afatinib	CSF	Imatinib	XBridge Shield RP18 (50 × 2.1 mm, 3.5 µm)	Acetonitrile (10 mmol/L ammonium hydroxide), water (1 mmol/L ammonium hydroxide), pH = 10.5	Isocratic (70:30)	ESI (+)	486.0 > 371.3	494.1 > 394.4	Not used	LLE	[60,117]
Afatinib	CSF	None	Inertsil ODS-2 (150 × 2.1 mm, 5 µm)	Water (0.1% ammonium acetate, pH = 8.5), acetonitrile, triethylamine	Isocratic (55:44:0.5)	Not applicable			254	SPE	[61]
Alectinib	CSF	Liquid chromatography–mass spectrometry was used.									[75]
Alectinib	CSF	Liquid chromatography–tandem mass spectrometry was used. Sample preparation consisted of deprot. with methanol.									[76]
Ceritinib	CSF	^13^C_6-_ceritinib	Acquity UPLC BEH C18 (50 × 2.1 mm, 1.7 µm)	Water (0.1% FA), methanol (0.1% FA)	Gradient	ESI (+)	558.0 > 433.0	564.3 > 438.9	Not used	Deprot.	[74,118]
Crizotinib	CSF	Liquid chromatography-tandem mass spectrometry was used.									[70]
Crizotinib	CSF	^2^H_5_,^13^C_2_-crizotinib	Discovery C18 (50 × 2.1 mm, 5 µm)	Water (0.3% FA), methanol (0.3% FA)	Gradient	ESI (+)	450.2 > 260.2	457.2 > 267.3	Not used	SPE	[71,119]
Crizotinib	CSF	Liquid chromatography-tandem mass spectrometry was used.									[72]
Crizotinib	CSF	No details of the employed analytical methodology are disclosed.									[73]
Dabrafenib	CSF	^2^H_9-_dabrafenib	XSelect HSS T3 (75 × 2.1 mm, 3.5 µm)	Water (2 mmol/L ammonium acetate, 0.1% FA), acetonitrile (0.1% FA)	Gradient	ESI (+)	520.1 > 292.0	529.1 > 316.2	Not used	Deprot.	[69,120]
Dasatinib	CSF	Carbamazepine	Nucleoshell C18 (150 × 3 mm, 2.7 µm)	Water (0.1% FA), methanol	Gradient	ESI (+)	488.17 > 232.1 488.17 > 193.1 488.17 > 161.0	237.1 > 194.2 237.1 > 165.1 237.1 > 121.1	Not used	Deprot.	[12]
Dasatinib	CSF	Quinoxaline	Atlantis C18 (150 × 4.6 mm, 5 µm)	Water (0.05% FA), acetonitrile (0.05% FA)	Gradient	ESI (+)	487.5	313.0	Not used	Deprot.	[27,121]
Dasatinib	CSF	^2^H_8_-dasatinib	Shim-Pack XR-ODSII (50 × 2 mm, 2.2 µm)	Water (0.1% FA), acetonitrile (0.1% FA)	Gradient	ESI (+)	488.0 > 401.0	496 > 406	Not used	NS	[28]
Erlotinib	CSF	Midazolam	C18 Luna (150 × 4.6 mm, 5 µm)	Acetonitrile, 5 mmol/L ammonium acetate	Isocratic (45:55)	ESI (+)	394.1 > 278.0 394.1 > 336.0	326.2 > 291.0	Not used	LLE	[45,122]
Erlotinib	CSF	Desmethyl erlotinib	Zorbax C18 (150 × 3 mm, 1.8 µm)	Acetonitrile, water (15 mmol/L ammonium acetate)	Gradient	ESI (+)	394.5 > 278.1	313.8 > 243.9	Not used	Deprot.	[46,123]
Erlotinib	CSF	Midazolam	Xterra octadecylsilica (50 × 2.1 mm, 3.5 µm)	Acetonitrile (0.1% FA), water (0.1% FA)	Isocratic (70:30)	ESI (+)	394 > 278	326 > 286.1	Not used	Deprot.	[47,116]
Erlotinib	CSF	Liquid chromatography–tandem mass spectrometry were used.									[48]
Erlotinib	CSF	OSI-597	Nova-Pak C18 (150 × 3.9 mm, 4 µm)	Acetonitrile, water (pH = 2.0)	Isocratic (60:40)	Not applicable			348	LLE	[50,124]
Erlotinib	CSF	None	Symmetry C18 (150 × 4.6 mm, 5 µm)	Acetonitrile, 0.05 mol/L aqueous potassium phosphate (0.2% triethylamine, pH = 4.8)	Isocratic (42:58)	Not applicable			345	LLE	[51,52,56,57,125]
Erlotinib	CSF	High-performance liquid chromatography was used.									[53]
Erlotinib	CSF	Deprot. with methanol-acetonitrile 1:4, v/v%. Liquid chromatography-tandem mass spectrometry was used.									[54]
Erlotinib	CSF	Liquid chromatography–tandem mass spectrometry was used.									[55]
Erlotinib	CSF	Liquid–liquid extraction and high-performance liquid chromatography with mass spectrometric detection was used.									[58]
Erlotinib	pleural effusate	None	Symmetry C18 (150 × 4.6 mm, 5 µm)	Acetonitrile, 0.05 mol/L aqueous potassium phosphate (0.2% triethylamine, pH = 4.8)	Isocratic (42:58)	Not applicable			345	LLE	[49,125]
Everolimus	Breast milk	^2^H_4_-everolimus	NS	NS	Gradient	ESI (+)	975.6 > 908.5	979.6 > 912.5	Not used	Online enrichment	[81,126]
Everolimus	Saliva	^13^C,^2^H_3_-everolimus	Sunfire C18	Water (20 mmol/L ammonium formate), methanol	Gradient	NS	NS	NS	Not used	LLE	[82,127]
Gefitinib	CSF	^2^H_8_-gefitinib	XTerra phenyl (50 × 4.6 mm, 5 µm)	Water (0.1% ammonia), acetonitrile	Isocratic (30:70)	APCI (+)	447.2 > 128.0	455.4 > 136.0	Not used	LLE	[39,40,42,43,114,128]
Gefitinib	CSF	Vandetanib	Intersil ODS3 (150 × 2.1 mm, 3 µm)	Water (0.02 mol/L ammonium acetate), acetonitrile.	Isocratic (70:30)	ESI (+)	447.2 > 128.1	475.6 > 112.0	Not used	LLE	[41,114]
Gefitinib	CSF	Erlotinib	Zorbax Eclipse XDB-C18 (150 × 4.6 mm, 5 µm)	Water (0.1% triethylamine, pH = 4.8), acetonitrile	Gradient	Not applicable			344	SPE	[44,129]
Gefitinib	CSF	^2^H_8_-gefitinib	Xterra octadecylsilica (50 × 2.1 mm, 3.5 µm)	Acetonitrile (0.1% FA), water (0.1% FA)	Isocratic (70:30)	ESI (+)	447.1 > 128.0	455.1 > 136.0	Not used	Deprot.	[45,130]
Gefitinib	pleural and peritoneal effusate	Liquid chromatography–tandem mass spectrometry was used.									[38]
Ibrutinib	CSF	^2^H_5_-ibrutinib	nLC EASY-Spray (50 cm)	NS	Gradient	Not specified (+)	441.2034 > 138.0900	446.2347 > 138.0900	Not used	Deprot.	[85]
Ibrutinib	CSF	Propranolol	Zorbax SB-C18 (150 × 2.1 mm, 5 µm)	Methanol, water (0.1% FA)	Gradient	ESI (+)	NS	NS	Not used	Deprot.	[86,131]
Icotinib	CSF	Liquid chromatography–tandem mass spectrometry was used.									[66]
Icotinib	CSF	Liquid chromatography–tandem mass spectrometry was used.									[67]
Imatinib	Breast milk	^2^H_8_-imatinib	Luna C18 (50 × 4.6 mm, 5 µm)	Methanol (0.1% FA), water (0.1% FA)	Gradient	ESI (+)	493.7	501.7	Not used	Deprot.	[20,132]
Imatinib	Breast milk	None	Luna C18 (50 × 4.6 mm, 5 µm)	Methanol (0.1% FA), water (0.1% FA)	Gradient	ESI (+)	494 > 394	Not used	Not used	Deprot.	[21,132]
Imatinib	Breast milk	Liquid chromatography–tandem mass spectrometry was used. Deprot. was employed as sample preparation.									[22]
Imatinib	Breast milk	No details of the employed analytical methodology are disclosed.									[23]
Imatinib	Breast milk	No details of the employed analytical methodology are disclosed.									[24]
Imatinib	Breast milk	No details of the employed analytical methodology are disclosed.									[25]
Imatinib	CSF	Carbamazepine	Nucleoshell C18 (150 × 3 mm, 2.7 µm)	Water (0.1% FA), methanol	Gradient	ESI (+)	494.27 > 394.2, 494.27 > 247.1, 494.27 > 217.2.	237.1 > 194.2 237.1 > 165.1 237.1 > 121.1	Not used	Deprot.	[12]
Imatinib	CSF	Liquid chromatography–tandem mass spectrometry was used.									[15]
Imatinib	CSF	^2^H_8_-imatinib	Symmetry Shield-RP8 (50 × 4.6 mm, 3.5 µm)	Methanol (0.05% ammonium acetate), water (0.05% ammonium acetate)	Isocratic (72:28)	APCI (+)	494.3 > 394.3	502.2 > 394.3	Not used	Deprot.	[16,115]
Imatinib	CSF	No details of the analytical methodology are disclosed.									[17]
Imatinib	CSF	None	ZirChromPDB-ZrO2 (50 × 4.6 mm, 3 µm)	Water (0.01 mol/L KH_2_PO_4_, 0.09 mol/L K_2_HPO_4_), methanol	Isocratic (60:40)	Not used			260	Deprot., online enrichment	[18]
Imatinib	CSF	No details of the analytical methodology are disclosed.									[19]
Imatinib	Leukocytes	Clozapine	Symmetry Shield-RP8 (50 × 4.6 mm, 3.5 µm)	Methanol (0.05% ammonium acetate), Water (0.05% ammonium acetate)	Isocratic (72:28)	Not applicable			261	SPE	[13]
Imatinib	PBMC	Quinoxaline	Atlantis T3 C18 (150 × 2.1 mm, 3 µm)	Water (0.05% FA), acetonitrile (0.05% FA)	Gradient	ESI (+)	493.8	313.0	Not used	Deprot.	[14,133]
Imatinib	semen	NS	CAPCELLPAK-C18	Water (2 mmol/L ammonium acetate, 0.05% TFA), acetonitrile-methanol 1:1 (0.05% TFA)	NS	ESI (+)	NS	NS	Not used	NS	[26]
Lapatinib	CSF	High-performance liquid chromatography–mass spectrometry was used.									[83]
Neratinib	CSF	Liquid chromatography–tandem mass spectrometry was used.									[84]
Nilotinib	CSF	NS	Nucleosil C18 HD (125 × 2 mm, 3.5 µm)	Acetonitrile, 0.05 mol/L aqueous potassium dihydrogenphosphate (pH = 4.03)	Isocratic (37:63)	Not applicable			258	Online enrichment	[29,134]
Nilotinib	CSF	No details of the analytical methodology are disclosed.									[30]
Nilotinib	CSF	^13^C,^2^H_3_-nilotinib	Acquity BEH C18 (50 × 2.1 mm, 1.7 µm)	NS	NS	ESI (+)	530.27 > 289.01	NS	Not used	Deprot.	[32,33,34,36]
Nilotinib	CSF	High-performance liquid chromatography and tandem mass spectrometry were used. The internal standard was ^2^H_6_-nilotinib.									[35]
Nilotinib	Pleural effusate	Liquid chromatography–tandem mass spectrometry was used.									[31]
Nintedanib	CSF	Carbamazepine	Nucleoshell C18 (150 × 3 mm, 2.7 µm)	Water (0.1% FA), methanol	Gradient	ESI (+)	540.26 > 113.1 540.26 > 70.2 540.26 > 42.2	237.1 > 194.2 237.1 > 165.1 237.1 > 121.1	Not used	Deprot.	[12]
Osimertinib	CSF	Liquid chromatography–tandem mass spectrometry was used.									[62]
Osimertinib	CSF	Sample pretreatment consisted of deprot. Liquid chromatography-tandem mass spectrometry was used.									[63]
Osimertinib	CSF	Liquid chromatography–tandem mass spectrometry was used.									[64]
Panobiostat	CSF	Carbamazepine	Nucleoshell C18 (150 × 3 mm, 2.7 µm)	Water (0.1% FA), methanol	Gradient	ESI (+)	350.2 > 158.2 350.2 > 143.1	237.1 > 194.2 237.1 > 165.1 237.1 > 121.1	Not used	Deprot.	[12]
Panobinostat	CSF	Liquid chromatography–tandem mass spectrometry was used.									[92]
Panobinostat	CSF	No details of the employed analytical methodology are disclosed.									[93]
Pazopanib	ascitic fluid	No details of the employed analytical methodology are disclosed.									[88]
Ponatinib	CSF	NS	Hypersil Gold PFP (100 × 2.1 mm, 1.9 µm)	Water (10 mmol/L formate ammonium buffer, 0.1% FA), acetonitrile (0.1% FA)	Gradient	ESI	NS	NS	Not used	Deprot.	[37,135]
Regorafenib	CSF	Carbamazepine	Nucleoshell C18 (150 × 3 mm, 2.7 µm)	Water (0.1% FA), methanol	Gradient	ESI (+)	483.09 > 288.1 483.09 > 270.1 483.09 > 202.0	237.1 > 194.2 237.1 > 165.1 237.1 > 121.1	Not used	Deprot.	[12]
Regorafenib	CSF	^2^H_5-_moxifloxacin	Kinetex F5 (50 × 4.6 mm, 2.5 µm)	Water (0.1% FA), methanol (0.1% FA)	Gradient	NS, (+) polarity	483.1 > 270.1	407.4 > 266.4	Not used	Deprot.	[89]
Ribociclib	CSF	Carbamazepine	Nucleoshell C18 (150 × 3 mm, 2.7 µm)	Water (0.1% FA), methanol	Gradient	ESI (+)	435.3 > 322.1 435.3 > 294.1 435.3 > 252.1	237.1 > 194.2 237.1 > 165.1 237.1 > 121.1	Not used	Deprot.	[12]
Ribociclib	CSF	^13^C_6-_ribociclib	Xbridge Amide (100 × 4.6 mm, 3.5 µm)	Acetonitrile, water (10 mmol/L ammonium formate, pH = 3.0)	Isocratic (75:25)	ESI (+)	435.3 > 367.2	441.3 > 373.2	Not used	Deprot.	[78,79,136]
Ribociclib	CSF	^2^H_6-_ribociclib	Polaris C8 (50 × 2.0 mm, 5 µm)	Water (0.1% FA), acetonitrile (0.1% FA)	Gradient	ESI (+)	435.2 > 252.1	441.2 > 252.1	Not used	Dilution, acidification, centrifugation	[80,137]
Sunitinib	ascitic fluid	No details of the employed analytical methodology are disclosed.									[88]
Tepotinib	CSF	Ultra-performance liquid chromatography was used.									[90]
Tepotinib	CSF	Liquid chromatography–tandem mass spectrometry was used.									[91]
Trametinib	CSF	^13^C_6-_trametinib	XSelect HSS T3 (75 × 2.1 mm, 3.5 µm)	Water (2 mmol/L ammonium acetate, 0.1% FA), acetonitrile (0.1% FA)	Gradient	ESI (+)	616.1 > 254.1 616.1 > 491.3	622.0 > 497.2	Not used	Deprot.	[69,120]
Vemurafenib	CSF	Sorafenib	XTerra C8 MS (250 × 4.6 mm, 5 µm)	Water (100 mmol/L glycine, pH = 9.0), acetonitrile	Isocratic (45:55)	Not applicable			249	Deprot.	[68,138]
Venetoclax	CSF	^2^H_8_-venetoclax	Atlantis C18 (50 × 2.1 mm, 3 µm)	Acetonitrile, water (0.1% FA)	Isocratic (55:45)	ESI (+)	868 > 321	876 > 329	Not used	Deprot.	[95,139]
Venetoclax	CSF	^2^H_8_-venetoclax	Atlantis C18 (50 × 2.1 mm, 3 µm)	Acetonitrile, water (0.1% FA)	Isocratic (55:45)	ESI (+)	868 > 321	876 > 329	Not used	LLE	[96,139]
Vismodegib	CSF	^2^H_5-_vismodegib	Betasil C18 (100 × 2.1 mm)	Water (0.1% FA), acetonitrile	Isocratic (40:60)	ESI (+)	421.1 > 139.2	426.1 > 139.1	Not used	SPE	[94,140]
Vorinostat	CSF	Carbamazepine	Nucleoshell C18 (150 × 3 mm, 2.7 µm)	Water (0.1% FA), methanol	Gradient	ESI (+)	265.16 > 232.1 265.16 > 77.1 265.16 > 55.1	237.1 > 194.2 237.1 > 165.1 237.1 > 121.1	Not used	Deprot.	[12]
Zanubrutinib	CSF	Tolbutamide	Zorbax SB-C18 (150 × 2.1 mm, 5 µm)	Methanol, water (0.1% FA)	Gradient	ESI (+)	NS	NS	Not used	Deprot.	[86,131]
Zanubrutinib	CSF	NS	Acquity BEH C18 (50 × 2.1 mm, 1.7 µm)	Water (0.15 FA), acetonitrile (0.1% FA)	NS	ESI (+)	NS	NS	Not used	Deprot.	[87]

## Data Availability

Not applicable.
